# Molecular and Morphological Data Improve the Classification of Plantagineae (Lamiales)

**DOI:** 10.3390/plants10112299

**Published:** 2021-10-26

**Authors:** Alexey Shipunov, José Luis Fernández-Alonso, Gustavo Hassemer, Sean Alp, Hye Ji Lee, Kyle Pay

**Affiliations:** 1 Department of Biology, Minot State University, Minot, ND 58707, USA; sean_alp@hotmail.com (S.A.); hyejix1115@hotmail.com (H.J.L.); kyle.pay@ndus.edu (K.P.); 2 Real Jardín Botánico, Plaza de Murillo 2, 28014 Madrid, Spain; jlfernandeza@rjb.csic.es; 3 Três Lagoas Campus, Federal University of Mato Grosso do Sul, Três Lagoas CEP 79610-100, Brazil; g.hassemer@ufms.br

**Keywords:** *Aragoa*, *Littorella*, *Plantago*

## Abstract

The tribe Plantagineae (Lamiales) is a group of plants with worldwide distribution, notorious for its complicated taxonomy and still unresolved natural history. We describe the result of a broadly sampled phylogenetic study of tribe. The expanded sampling dataset is based on the *trn*L-F spacer, *rbc*L, and ITS2 markers across all three included genera (*Aragoa*, *Littorella* and *Plantago*) and makes this the most comprehensive study to date. The other dataset uses five markers and provides remarkably good resolution throughout the tree, including support for all of the major clades. In addition to the molecular phylogeny, a morphology database of 114 binary characters was assembled to provide comparison with the molecular phylogeny and to develop a means to assign species not sampled in the molecular analysis to their most closely related species that were sampled. Based on the molecular phylogeny and the assignment algorithm to place unsampled species, a key to sections is presented, and a revised classification of the tribe is provided. We also include the description of new species from North America.

## 1. Introduction

The tribe Plantagineae (Lamiales) consists of three diverse genera of plants: the worldwide distributed plantains (*Plantago*), the small aquatic *Littorella*, and a genus of northern Andean shrubs (*Aragoa*). This group is notorious for its complicated taxonomy, the difficulty of species identification, and an evolutionary trend of morphological reduction and simplification [1,2,3,4].

Plantains (ribworts), *Plantago* L., grow almost everywhere in the world, except for Antarctica and tropical wet forests [2,5]. Morphologically, they bear an unusual combination of characters [6]: sympetalous 4-merous non-showy flowers developing into circumscissile capsule-like fruit (pyxidium), and monocot-like leaves with arcuate or parallel venation, usually borne in a rosette. Even in the eighteenth century, when only sixteen species were described in *Plantago* [7], botanists mentioned the remarkable similarity between species, and many collections today still hold a significant amount of incorrectly determined specimens. Plantains exhibit reduction of both vegetative and generative characters. A few species have a branched stem and “dicotyledonous” leaves. Recent research [8] demonstrated how flower reduction could occur within Antirrhinum-*Plantago* lineage, in which evolution towards anemophily resulted in significant morphological convergence with graminoid monocots. *Plantago* likely underwent a rapid evolution [9] and relatively recent diversification [10]. *Plantago* includes both species with broad distribution and local endemics or near endemics. On many remote ocean islands, for example, there are unique species of *Plantago* [11,12]. Several new *Plantago* species have recently been described, and most of them are rare and narrowly distributed (e.g., [13,14,15]). All of the above makes *Plantago* a group of plants of considerable interest for biodiversity conservation [5]. The genus also has a number of complicated nomenclatural problems remaining to be solved [16,17,18,19].

Aquatic *Littorella* P.J.Bergius [20] comprises three species with considerably disjunct distribution; they grow in shallow waters of North America Great Lakes, Patagonia, and Northern Europe. These plants are morphologically similar to *Plantago*, and Rahn [2] synonymized the two genera. However, when molecular data became available [21], *Plantago* and *Littorella* were shown as sister clades, and since then nearly all authors preferred to keep the tradition of recognizing the two genera as separate.

Contrary to *Plantago* and *Littorella*, the woody shrubs in the genus *Aragoa* Kunth [22] are the local endemics of páramo in Colombia (one species also in Venezuela). The genus was considered unplaced within the Scrophulariaceae [1] until the molecular analyses revealed it to be a sister group of the *Plantago* + *Littorella* clade [23]. *Aragoa* is relatively speciose, containing more than 20 described species and also several hybrid species [1,24], whereas no hybrid species have been described in two other *Plantago* or *Littorella*. Intensive hybridization might explain the rapid radiation and speciation in this genus [25]. Flowers of *Aragoa* are likely animal-pollinated [1] but: actinomorphic; leaves are reduced, similarly to two other genera of the group [26].

*Plantago*, *Littorella* and *Aragoa* form a clade that is well-supported both morphologically and molecularly [3,23,26], which we call hereafter the tribe Plantagineae Dumort [27]. Multiple detailed morphology-based works were published about Plantagineae; most important are [1,2,28,29,30]. However, a comprehensive molecular study based on broadly sampling this whole group is still absent. This situation has shown signs of improvement lately ([31]), and several publications which cover *Plantago* subg. *Plantago* (e.g., [4,12]) and *Plantago* subg. *Coronopus* ([14,19,32]) are now available. Some recent regional works ([33,34,35]) have also improved our knowledge of subg. *Plantago*. However, no recent molecular works focusing on *Aragoa* diversity exist, and since [3], nothing significant has been published about the molecular taxonomy specific to *Plantago* subgg. *Bougueria*, *Psyllium* s.str. and *Albicans*.

The broadest molecular phylogenies currently available are the works [3,21], which included 59 and 27 species, respectively. The whole group, however, is estimated to include ca. 250 species. The GenBank database as of June 2020 contains only about 140 species entries (and this does not account for possible synonymy).

Therefore, we consider Plantagineae a largely under-studied group, especially in the molecular aspect. We here continue the line of [3] and employ similar markers but aim for greater species coverage, with the ultimate goal to assess all the described species of the group and obtain a detailed picture of their relationships. Together with molecular characters, we employ morphological characters identified in [2] and morphometric characters from samples used in molecular studies. Our main objectives were: (1) sample species which were not included in previous phylogenies; (2) use molecular and morphological data as a basis to putatively place species which were not previously classified into subgenera and sections; (3) present a taxonomic key for *Plantago* subgenera and sections and (4) present taxonomic novelties in the genus *Plantago*.

## 2. Materials and Methods

### 2.1. Plant Material

Due to the large number of species in the group and worldwide distribution, broad sampling is challenging. Therefore, while some of our samples were collected into silica gel from living plants, the majority of work (94%) involved tissue samples taken (with the kind permission of herbarium curators) from plants collected years ago (see Table S1).

Using herbarium samples poses some restrictions. While the purity and concentration of DNA in Plantagineae do not significantly decrease with time ([36]), the quality of sequences heavily depends on sample age, collection methods, and the nature of the amplified DNA region. We have now sequences from 220 species (including 192 *Plantago* species). Data from 86 species have been taken from public databases. In all, we were able to increase the amount of available information three-fold (four-fold in *Aragoa*). Due to common problems with voucher identification [37], we always used our own identification and, if there was a choice, preferred to use our own samples.

In this paper, we followed the “appropriate citation of taxonomy” (ACT) principle, that is to “to ensure that common elements of taxonomic papers, generally considered de facto citations by taxonomists but not by [citation indexes], are presented in a format that is considered a valid citation by [citation indexes]” ([38]). Thus, we cited in the References names of the most important supra-species groups ([39]).

### 2.2. DNA Extraction and Sequencing

DNA extraction performed using multiple standard protocols, but soon after the start of the project (2011), we decided to exclusively use the NUCLEOSPIN Plant II Kit (MACHEREY-NAGEL GmbH & Co., KG, Düren, Germany) which appeared to provide a good trade-off between efficiency and simplicity. We improved this protocol in several respects, e.g., increased the lysis time to 30 min and used a thermomixer on slow rotation speed (350 rpm) instead of a water bath. To assess DNA quality, we used Nanodrop 1000 Spectrophotometer (Thermo Scientific, Wilmington, DE, USA), which estimates concentration and purity (the 260/280 nm ratio of absorbance) of samples. Typically, 1.4 ratio was enough to guarantee PCR amplification of smaller markers; the average ratio was 1.7. Lower DNA quality was typically obtained from samples collected in humid environments. Especially low was the quality of our *Aragoa* samples. We speculate that this might be due to the use of drying cabinets; indeed, when we dried our own *Aragoa* samples in room conditions, they yielded high-quality DNA.

Short DNA markers are best to amplify for herbarium samples [40]; therefore, our first choices were the nuclear ITS2 and plastid *trn*L-F spacer and *rbc*L genes. We amplified them following recommendations and protocols from Barcoding of Life ([40]). Several samples were sequenced with the direct help of Barcoding of Life (“SAPNA” project); they also provided us with sequences of mitochondrial COI and plastid *mat*K markers. ITS sequences were always checked against the GenBank database to exclude potential contamination with DNA from other plant groups or fungi.

Our PCR reaction mixture for most samples had a total volume of 20 µL which contained 5.2 µL of PCR Master Mix (components mostly from Platinum DNA Taq Polymerase, Thermo Fisher Scientific, Waltham, Massachusetts, USA) supplied with Platinum DNA Taq Polymerase), 1 µL of 10 µM forward and reverse primers, 2 µL of DNA solution from the extraction and 10.8 µL of MQ purified water (obtained from a Barnstead GenPure Pro system, Thermo Scientific, Langenselbold, Germany) in the TBT-PAR water mix ([41]). The latter was developed to improve amplification from the herbarium samples. Thermal cycler programs for most samples were 94 ${}^{\circ }$C for 5 min, then 35 cycles of 94 ${}^{\circ }$C for 1 min; 50–52 ${}^{\circ }$C (depending on the primer) for 1 min, 72 ${}^{\circ }$C for 2 min, and finally 72 ${}^{\circ }$C for 10 min. PCR products were sent for purification and sequencing to Functional Biosciences, Inc. (Madison, Wisconsin); and sequenced there in both directions using a standard Sanger-based protocol. Sequences were obtained, assembled, and edited using Sequencher™ 4.5 (Genes Codes Corporation, Ann Arbor, MI, USA).

### 2.3. Phylogenetic Analyses

Analysis followed the “Ripeline” workflow. This workflow is a collection of UNIX shell and R ([42]) scripts that automate steps related to sequence selection, quality checking, alignments, gap coding, concatenation, and phylogenetic tree production. Ripeline involves multiple pieces of software, for example, AliView (San Francisco, CA, USA) [43], MUSCLE (San Francisco, CA, USA) [44], APE (San Francisco, CA, USA) [45], MrBayes (San Francisco, CA, USA) [46], ips (San Francisco, CA, USA) [47], shipunov (San Francisco, USA) [48], and phangorn (San Francisco, CA, USA) [49].

Using Ripeline, we were able to obtain maximum likelihood (ML), Bayesian (MB), and maximum parsimony (MP) phylogenetic trees. Maximum likelihood analyses were run RAxML ([50]) with 10,000 bootstrap replicates and R ips package ([47]). We used a GTR+G+I model based on model testing with R phangorn package ([49]). Bayesian analyses were run through the combination of MrBayes 3.2.6 ([46]), and R shipunov package ([48]). MCMC chains were run for 10,000,000 generations, sampling every 10th generation resulting in 1,000,000 trees. The first 25% of trees were discarded as burn-in, and the remaining trees were summed to calculate the posterior probabilities; we used strict consensus tree. The convergence of Bayesian analyses was controlled using the standard deviation of frequencies across runs, and the potential scale reduction factor, PSRF ([46]). Maximum parsimony analyses were run with the help of R phangorn package ([49]) using parsimony ratchet ([51]) with 2000 iterations and then 1000 bootstrap replicates. All trees were rooted with *Veronicastrum virginicum* (L.) Farw. and *Tetranema roseum* (M.Martens & Galeotti) (= *Tetranema mexicanum* Benth.) as outgroups, or with *V. virginicum* alone. To assess the congruence between chloroplast, mitochondrial and nuclear data, we used the CADM test ([52]).

We used several multivariate methods to assess phylogenetic trees: for example, to visualize the general structure of phylogenetic trees in the compact way, we employed multidimensional scaling on cophetetic distances between tree tips and created “density surfaces” (density of points was estimated with 2D binned kernel).

Since all single marker trees were concordant, we used the super-matrix approach. Our first dataset included multiple markers available from public databases and our sequencing, which is plastid *rbc*L, *trn*L-F, *mat*K, and also nuclear ITS2 and mitochondrial COI. We call this dataset “broad” since it is relatively rich in data but has a limited sampling along the species dimension (87 entries and 4188 bp, including 656/497/561/1565/909 bp in COI, ITS2, *rbc*L, *trn*L-F and *mat*K, respectively). The second dataset was made with the most extensive species coverage but included only ITS2 and *trn*L-F data, which is originated mostly from our sequencing. Below, we designate it as “tall” (273 entries, including some subspecies and forms and 2062 bp length, including the same ITS2 and *trn*L-F fragment lengths as above). The amount of missing data in these two datasets was 23% and 18%, respectively.

### 2.4. Morphology

Ripeline is also capable of using morphological characters, and we employed the updated and expanded morphological dataset from [2] to make the morphological dataset. To convert this dataset into digital, we applied optical character recognition, cleaned the results, converted tables into spreadsheets and merged them. We also added characters of seed sculpture ([53,54]) to the characters used in [2], and expanded the dataset with species absent in the last work. In total, our morphological matrix has 114 characters; all character states are binary. To make a combined molecular and morphological matrix, we multiplied [55] the morphological dataset several times in order to achieve the approximate parity between of molecular and morphological characters.

We measured seven additional morphometric characters of *Plantago* (petiole, leaf, spike, and scape lengths, maximal leaf width, presence of taproot, and presence of gaps in the inflorescence) on 405 herbarium samples, the same samples which were used in DNA extraction.

Using these three datasets (morphological, morphometric and DNA), we were able to perform a broad spectrum of statistical analyses, for example, Procrustes analysis of the correspondence between molecular and morphological information ([56,57]) and nearest neighbor machine learning ([58]). First method helped us to understand the comparative roles of molecular and morphological characters, second allowed to place approximately the under-studied taxa. To help with the construction of dichotomous keys for identification of *Plantago* sub-groups, we employed recursive partitioning ([59]), a machine learning technique which creates the classification trees in a way similar to identification keys ([32,60]). To find patterns in the character distribution, we compared leaf shapes (from morphometric dataset) by subgenera, sections, and macro-regions and used chi-square tests to assess results. To determine the significance of morphological characters in respect to the molecular phylogenetic tree (“molecular weight”), we used the average or maximum Spearman correlation between morphological matrices and phylogenetic trees based either on a “tall” dataset or molecular-morphological dataset.

All datasets, scripts, and phylogenic trees used in the preparation of this publication are available from the first author’s Open Repository here: http://ashipunov.info/shipunov/open/plantago.zip (accessed on 17 October 2021). Ripeline is available on Github: https://github.com/ashipunov/Ripeline (accessed on 17 October 2021). We encourage readers to reproduce our results and develop our methods further. All sequences were deposited into GenBank.

## 3. Results

### 3.1. Plantagineae in General

In total, we generated 296, 15 and 158 new sequences of ITS, *rbc*L and *trn*L-F, respectively.

**Figure 1:**
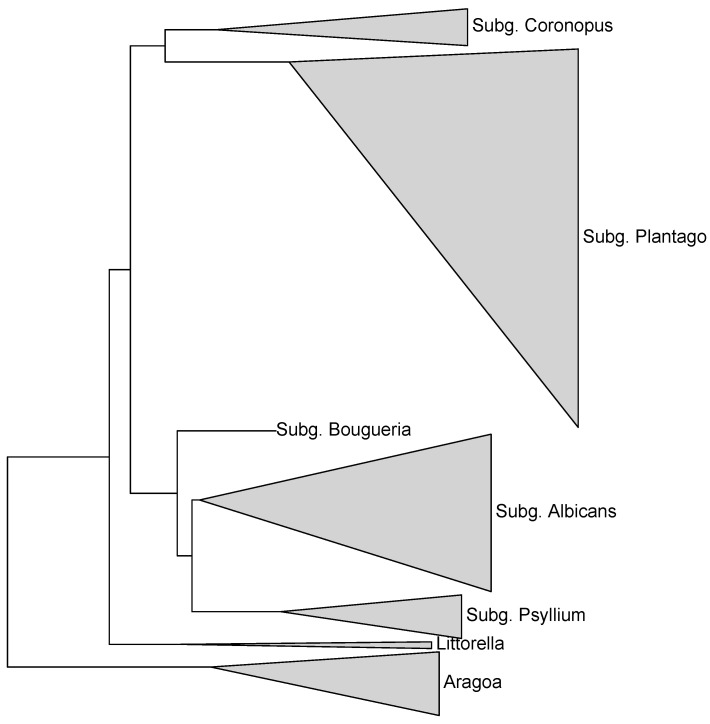
Phylogeny of the Plantagineae: general arrangement of clades (genera and subgenera). Each triangle is the result of concatenation applied to the branches of the Bayesian (MB) phylogenetic tree; internal branches are collapsed and triangle size represents number of species. Names beginning with “subg.” are subgenera of *Plantago*.

**Figure 2:**
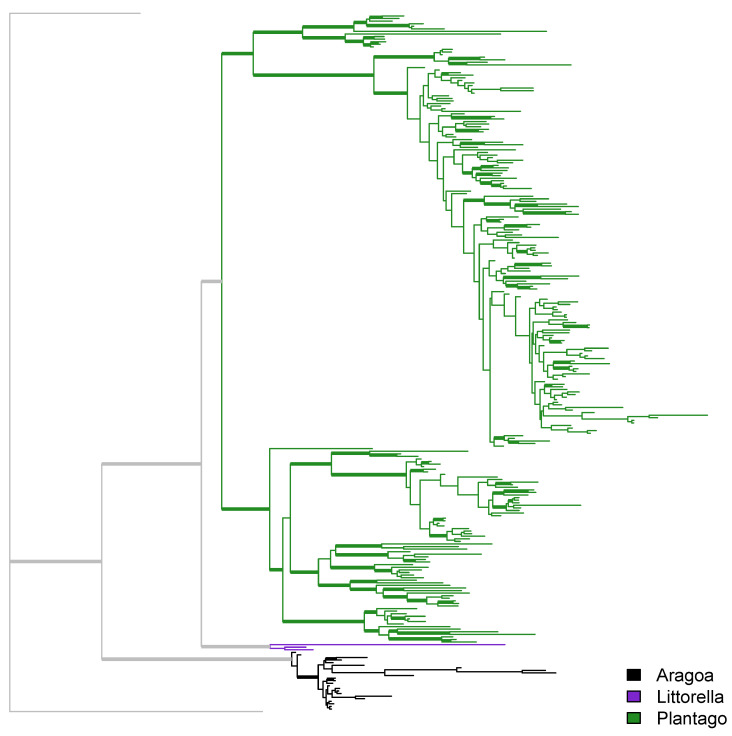
Overview of the MB tree based on the “tall” dataset. Branches with support > 90% are thickened.

**Figure 3:**
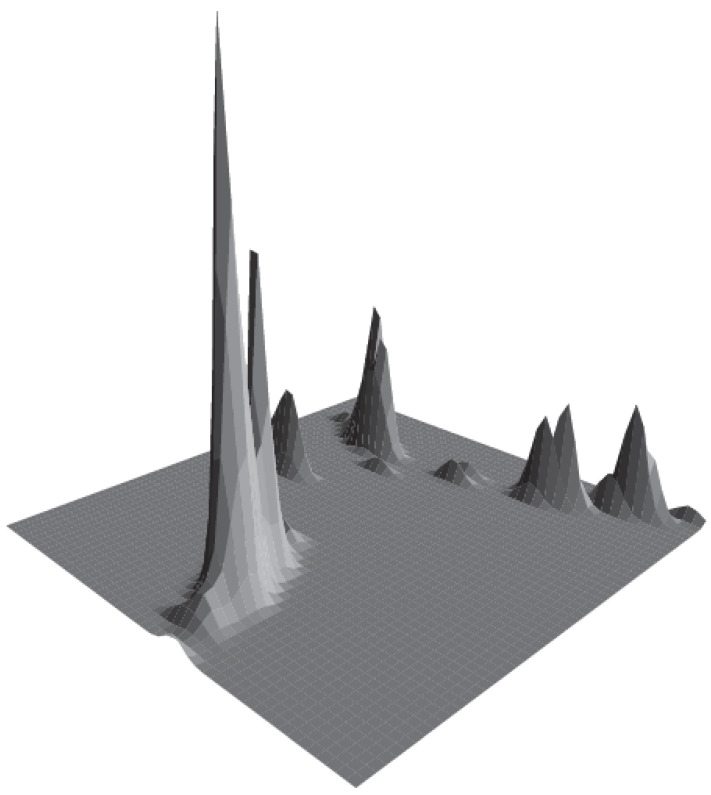
Density surface of the cophenetic space, based on the MB trees from the “tall” dataset. This surface is the result of multidimensional scaling of the cophenetic distances between tree tips; density of points estimated with 2D binned kernel. “Ridges” reflect areas with the highest density and correspond well with three major subgeneric divisions of *Plantago*. Note the three-fold structure of the “phylogenetic surface”: tallest corresponds with *Plantago* subg. *Plantago*, close and behind it, is *Plantago* subg. *Coronopus* whereas *Plantago* subgg. *Psyllium* and *Albicans* are the rightmost.

**Figure 4:**
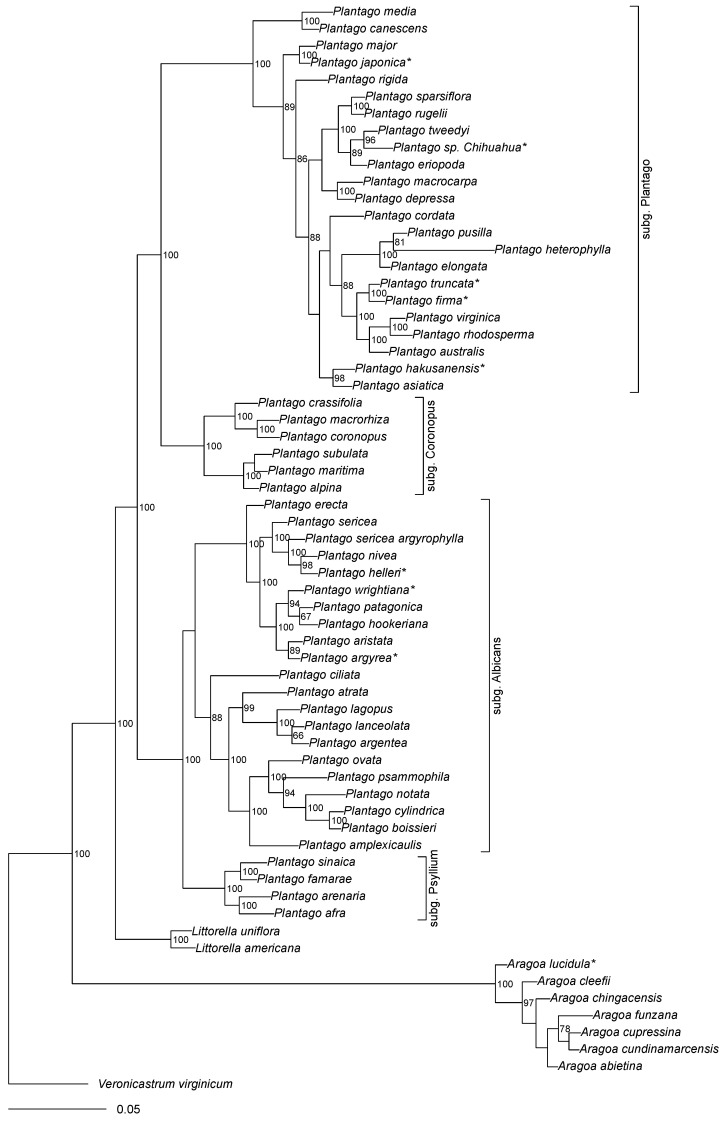
The phylogeny of Plantagineae obtained from the “broad” dataset (based on MB trees). Stars (*) mark species which have not been barcoded before. Numbers on nodes are Bayesian posterior probabilities (%). Names started with “subg.” are subgenera of *Plantago*.

**Figure 5:**
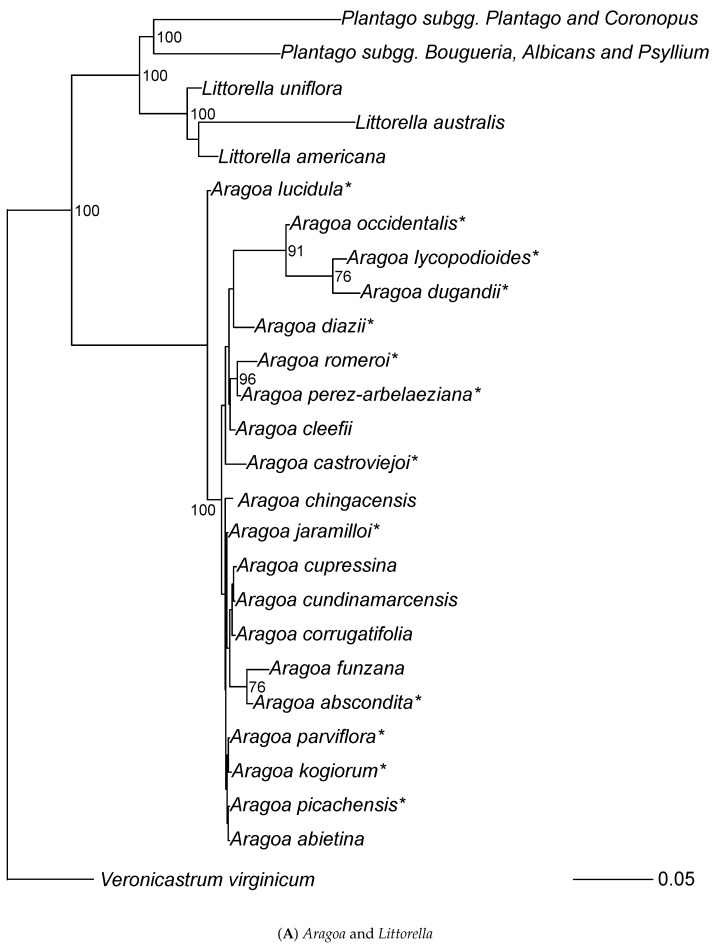

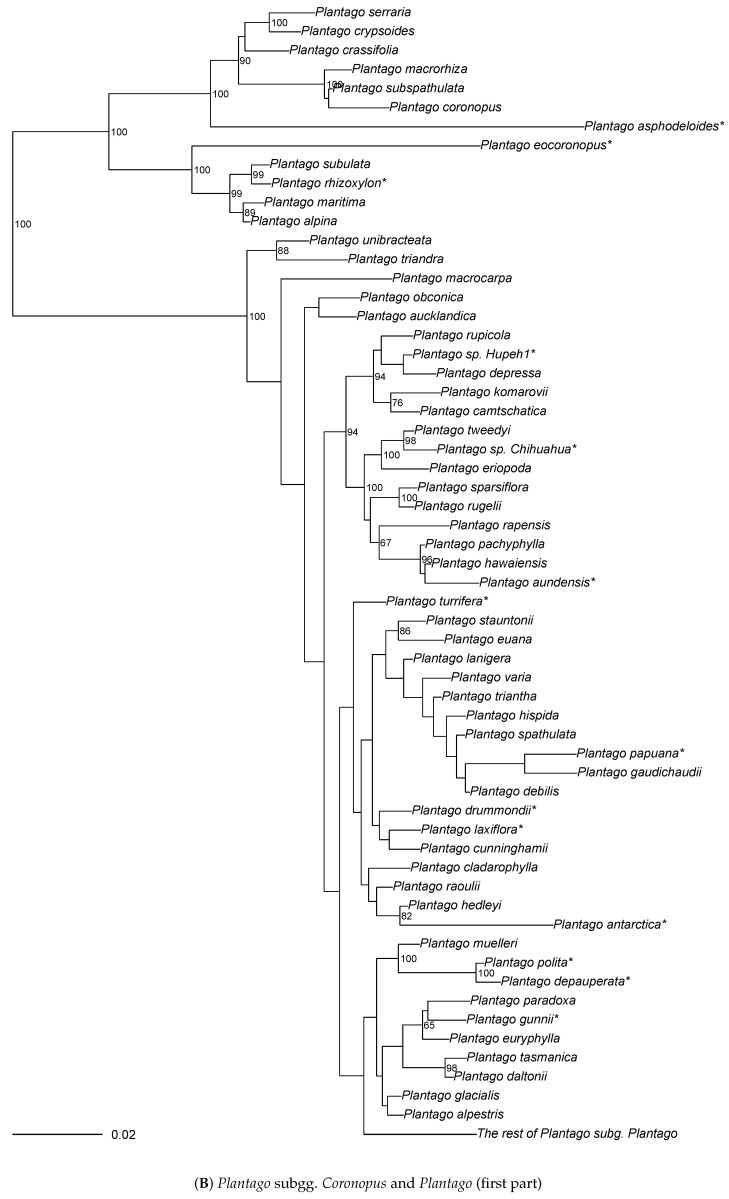

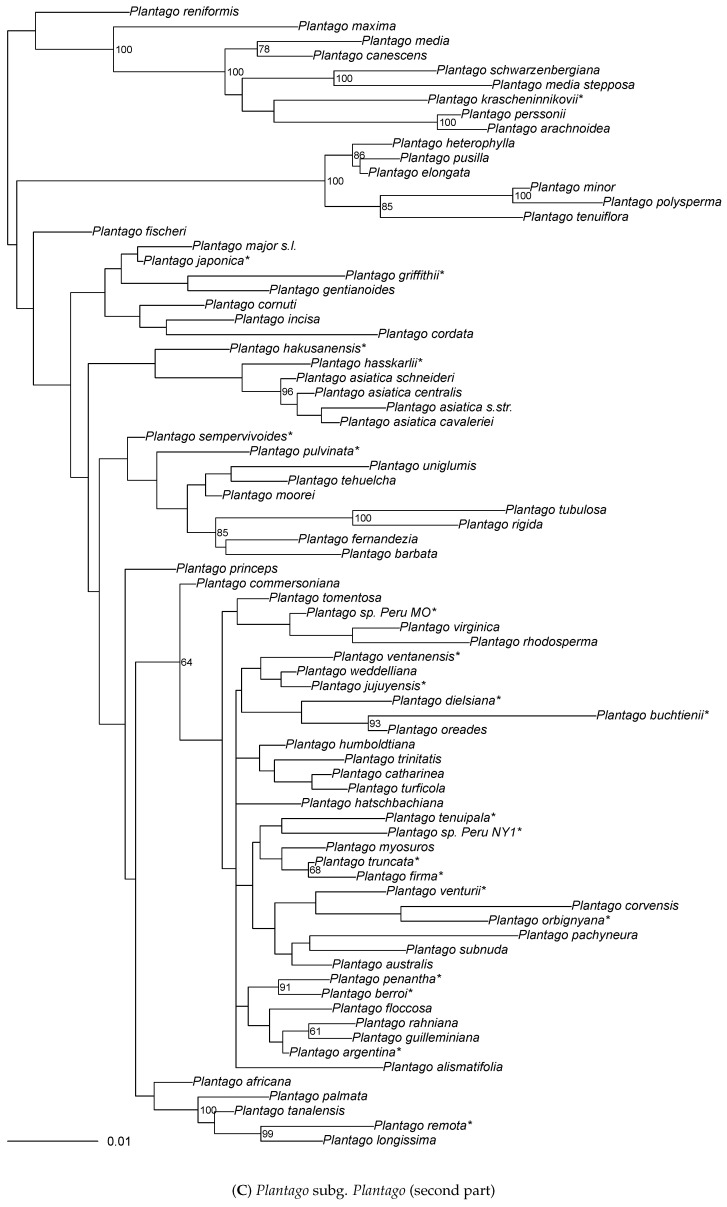

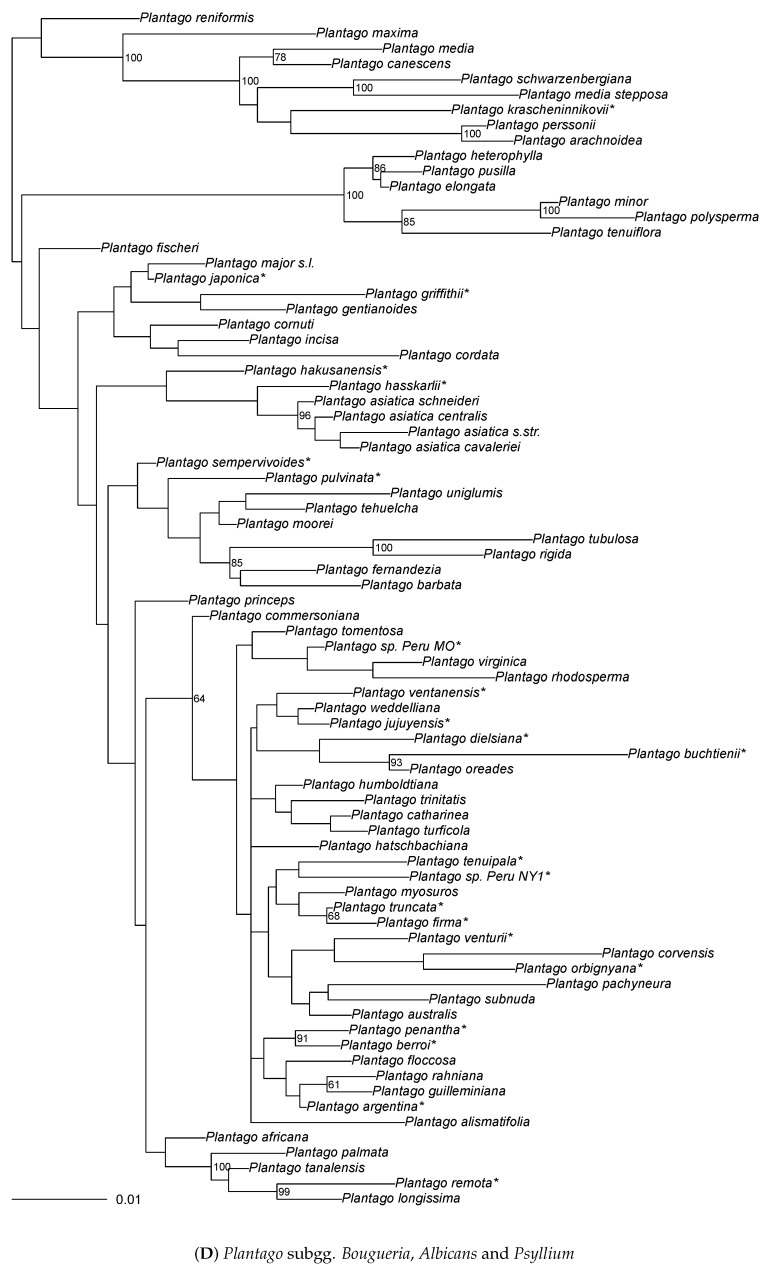
The phylogeny of Plantagineae obtained from the “tall” dataset (based on MB trees). (**A**) *Aragoa* and *Littorella*. (**B**) *Plantago* subgg. *Coronopus* and *Plantago* (first part). (**C**) *Plantago* subg. *Plantago* (second part). (**D**) *Plantago* subgg. *Bougueria*, *Albican*s and *Psyllium*. Stars (*) mark species which have not been barcoded before. Numbers on nodes are Bayesian posterior probabilities (%).

All trees based on “broad” and “tall” datasets returned the highly supported (about 100% bootstrap and 100 BPP) topology (*Aragoa*, (*Littorella*, *Plantago*)) (Figure 1), typically with the longest branch leading to *Aragoa*. As our maximum parsimony (MP) trees generally do not differ from Bayesian (MB) trees, we hereafter present the results (Figure 1, Figure 2, Figure 3, Figure 4 and Figure 5) based mostly on the second type of analysis.

CADM test for the congruence between *trn*L and ITS parts of “tall” supermatrix returned the high Kendall concordance value (W = 0.85147994), the null hypothesis of incongruence was rejected with *p*-value 0.00009999 (Chi-squared = 63690.69985174). CADM tests for the congruence between COI, ITS and *rbc*L parts of “broad” supermatrix also returned the high Kendall concordance values (W = 0.60137286 and 0.71073409, respectively), the null hypotheses of incongruence were both rejected with *p*-values 0.00109989 (Chi-squared = 4602.90790653) and 0.00009999 (Chi-squared = 5439.95871762), respectively.

### 3.2. Aragoa

Generally, the support of subclades is low in *Aragoa* (Figure 2). The most stable is a placement of *Aragoa* lucidula S.F.Blake as a sister to all other studied *Aragoa* species. Within the rest of the subtree, the majority of species make one clade with subdivisions mostly without high support. Morphologically outstanding (with relatively broad leaves) *A. dugandii* Romero forms a clade with *A. lycopodioides* Benth. and *A. occidentalis* (all branches here are longer than in other parts of *Aragoa* tree). The other unusual species (with long, yellow corolla tubes), *A. perez-arbelaeziana* Romero, forms a clade with *A. romeroi* Fern.Alonso (Figure 4 and Figure 5A).

### 3.3. Littorella

Three (two in the “broad” dataset) species of *Littorella* make a stable group where European *L. uniflora* (L.) Asch. is sister to American *L. americana* Fernald and *L. australis* Griseb. ex Benth. & Hook.f. (Figure 4 and Figure 5A).

### 3.4. Plantago in General

There is high support for three major subdivisions of *Plantago*, which correspond with subgeneric rank: the topology is robustly ((*Bougueria*,(*Psyllium* s.str., *Albicans*)),(*Coronopus*, *Plantago*)). *Plantago* subgg. *Plantago*, *Coronopus*, and *Psyllium* s.l. form a “three-ridge” phylogenetic density surface (Figure 3).

### 3.5. Plantago subg. Plantago

Trees originating from the “broad” dataset contain many highly supported clades whereas “tall” trees have reliable support only for some terminal clades (Figure 2, Figure 4 and Figure 5B–D).

One of these highly supported groups consists of *Plantago media* L., *Plantago canescens* Adams, *P. arachnoidea* Schrenk ex Fisch. & C.A.Mey., *P. krascheninnikovii* C.Serg., *P. maxima* Juss. ex Jacq., *P. perssonii* Pilg. and *P. schwarzenbergiana* Schur. A tetraploid, xeromorph variant of *P. media* described as *P. urvillei* Opiz (*P. media* subsp. stepposa (Kuprian.) Soó) typically does not branch with *P. media* s.str. *Plantago krascheninnikovii* from the Urals is habitually similar to the inland forms of *P. maritima* L.—(however, it lacks the key feature of *Plantago* subg. *Coronopus*, i.e., pilose corolla tube). On our trees, it groups with *P. arachnoidea* from Central Asia. Chinese *Plantago perssonii* Pilg. (including *P. lorata* (J.Z.Liu) Shipunov described from Central Asia: [61]) robustly groups with *P. arachnoidea*. Morphologically unusual *P. reniformis* Beck from Balkans frequently also groups here with low support.

Another highly supported group consists of species from *Plantago* sect. *Micropsyllium*: palearctic *P. polysperma* Kar. & Kir., *P. tenuiflora* Waldst. & Kit., and nearctic *P. elongata* Pursh, *P. heterophylla* Nutt. and *P. pusilla* Nutt. Plants from Öland (first described as separate species *P. minor* Fr.) does not group with *P. tenuiflora* but group instead with *P. polysperma*.

The less stable but relatively consistent group forms around polymorphic *P. asiatica* L. from mainland China and Japan, including *P. schneideri* Pilg., *P. centralis* Pilg., and *P. cavaleriei* H.Lév. We found typical *P. asiatica* on Hawaii Island, thus extending the range of this East Asian species to the mid-Pacific. However, all “*P. asiatica*” from mainland USA are either *P. major* or *P. rugelii* Decne. ([62,63]).

*Plantago hakusanensis* Koidz. is a Japanese alpine endemic species with a distinct morphology. On our trees, it is branched closely to *P. asiatica*. In PE herbarium, we discovered the Yunnan sample labeled as “*Plantago zhongdainensia*” (nomen nudum), which morphologically might be considered similar to both *P. hakusanensis* and *P. asiatica*. Unfortunately, DNA data is not available from this sample. Proximal to *P. asiatica* is also morphologically distinct *P. hasskarlii* Decne. from Java mountains. Another species from Southeast Asia, *P. incisa* Hassk., groups outside of *P. asiatica* clade(s).

An unusual sample “*Plantago* sp. Hupeh1” was collected from China. It has morphological similarities with *P. densiflora* J.Z.Liu (synonymized with *P. asiatica* in the “Flora of China,” [64]), but has 4–6 large black seeds and large fruits which is not in agreement with *P. densiflora* protologue. On our trees, it groups with *P. depressa* Willd. and allies (e.g., *P. komarovii* Pavlov and *P. camtschatica* Link). Besides, on “broad” trees, *P. depressa* robustly groups together with *P. macrocarpa* Cham. & Schltdl.; this grouping is also present on “tall” trees with less support.

American *P. eriopoda* Torr., *P. rugelii*, *P. sparsiflora* Michx., and *P. tweedyi* A.Gray are robustly supported as a clade on “broad” trees. Two samples collected in Chihuahua desert (BRIT) from northern Mexico also belong to this clade. They cluster together with *P. eriopoda* and *P. tweedyi* but morphologically are very distinct (see below).

Morphologically, *Plantago rugelii* is similar to *P. major*, but they do not group on any tree. *Plantago major* in turn, does not branch closely to morphologically similar *P. asiatica*, which was pointed out in [4]. Instead, *P. major* s.l. groups with *P. japonica* Fr. & Sav., *P. cornuti* Gouan, *P. gentianoides* Sibth. & Sm. and *P. griffithii* Decne., albeit with low support. Sequences from polyspermous form of *P. major* [65] described as *P. uliginosa* F.W.Schmidt, are identical to the typical *P. major*. *Plantago griffithii*, which is frequently considered a form of *P. gentianoides*, groups with the *P. gentianoides* s.str. on our trees, but this grouping is unstable.

*Plantago pachyphylla* A.Gray and *P. hawaiensis* (A.Gray) Pilg. (both from Hawaii) group together, and also with *P. aundensis* P.Royen from New Guinea. Alpine form of *P. pachyhylla* from Kauai (labeled in HUH as “*Plantago nubicola* Tessene,” nomen nudum) clusters outside of *P. hawaiensis* + *P. pachyphylla* from Hawaii Island.

Two New Zealand species, *P. triandra* Berggr. and *P. unibracteata* Rahn, always cluster together outside of the rest of *Plantago* subg. *Plantago*.

Our phylogenic trees, especially from the “tall” dataset, also provide the primary ground for the placement of little-studied or previously molecularly not studied forms, for example, for *P. laxiflora* Decne. This South African species is morphologically unusual for the region and groups outside of African species. Other African and Madagascan species, i.e., *P. africana* Verdc., *P. longissima* Decne., *P. palmata* Hook.f., *P. remota* Lam. and *P. tanalensis* Baker, tend to group together with low support.

Most of *Plantago* sect. *Virginica* ([66]) species do not group with high support. However, we note that Andean *P. oreades* Decne. always branches outside of the *P. australis* Lam. group. Another Peruvian form from this section was listed by Knud Rahn (MO herbarium note) as a possible new species; on our trees, it groups with different members of the section, including South American *P. tomentosa* Lam. The second “unknown” from Peru, *Plantago* sect. *Virginica* sample from NY with a long stem (unusual in *Plantago* subg. *Plantago*) frequently groups with *P. tenuipala* (Rahn) Rahn from Columbia.

*Plantago firma* Kunze ex Walp. was typically considered as strictly Chilean species, but we have found its samples collected in Peru (USM). All Chilean and Peruvian *P. firma* samples robustly group together, and then with another species, bipolarly distributed *P. truncata* Cham. & Schltdl.

While there is a little confidence among branches that belong to the rest of *Plantago* sect. *Virginica*, we were able to place in that group those species which have not been sampled before, namely *P. argentina* Pilg., *P. berroi* Pilg., *P. buchtienii* Pilg., *P. dielsiana* Pilg., *P. floccosa* Decne., *P. jujuyensis* Rahn, *P. orbignyana*, *P. penantha* Griseb., *P. tenuipala* (Rahn) Rahn, *P. ventanensis* Pilg. and *P. venturii* Pilg.

Most of Knud Rahn’s Oliganthos species (this group includes *P. barbata* G.Forst., *P. correae* Rahn, *P. pulvinata* Speg., *P. sempervivoides* Dusén, and *P. uniglumis* Wallr. ex Walp.) were sequenced for the first time. On our “tall” trees, the group does not have high support but clusters together with *P. moorei* Rahn, *P. tehuelcha* Speg. and *P. fernandezia* Bertero ex Barnéoud, all from Southern Cone and surrounding islands.

Most of the Australian species form a low-supported but relatively stable grade; here we were able to place some under-researched species: *P. antarctica* Decne., *P. depauperata* Merr. & L.M.Perry, *P. drummondii* Decne., *P. gunnii* Hook.f., *P. polita* Craven (New Guinea) and *P. turrifera* B.G.Briggs & al.

### 3.6. Plantago subg. Coronopus

All topologies supported the subdivision of sects. Maritima and Coronopus (Figure 4 and Figure 5B). Within *Plantago* sect. *Maritima*, we were able to place with confidence the rare Central Asian *P. eocoronopus* Pilg. (as a sister to the whole group) and North African *P. rhizoxylon* Emb. We detected the presence of the “true” *P. maritima* in South Africa (PRE herbarium); these samples are molecularly not different from the rest of *P. maritima*.

Macaronesian *P. asphodeloides* Svent. is the sister to other species from *Plantago* sect. *Coronopus*, and North African *P. crypsoides* Boiss. is sister to Mediterranean *P. serraria* L.

### 3.7. Plantago subgg. Bougueria, Albicans and Psyllium

Within this highly supported group (character abbreviations are explained in the Support Table S3, Figure 4 and Figure 5D), *P. nubicola* (Decne.) Rahn (which sometimes regarded as a separate genus Bougueria) always as sister to all other species where the following topology is prevalent: (Psyllium s.str., (“American clade,” “*Plantago ciliata* clade,” “Mediterranean clade”)); these we will describe in detail below.

Psyllium forms a robust, relatively long branch that split between mostly annual species with non-linear bracts (e.g., *P. squarrosa* Murray) and mostly perennial, woody species with narrow bracts (e.g., *P. arborescens* Poir.).

“*Plantago ciliata* clade” on “tall” trees is sister to “American clade”, whereas on “broad” trees *P. ciliata* Desf. is sister to “Mediterranean clade” (with lower support). This clade includes *P. ciliata* and two successfully sampled species from the *Plantago* sect. *Hymenopsyllium*, *P. cretica* L. and *P. bellardii* All.

“American clade” is, in essence, Rahn’s *Plantago* sect. *Gnaphaloides*. *Plantago erecta* E.Morris is variably at the base of this group, and *P. helleri* Small branches close to the southern *P. nivea* Kunth. The rest of North American species form a stable clade (which therefore roughly corresponds with Rahn’s ser. *Gnaphaloides*), where *P. aristata* Michx. and *P. argyrea* E.Morris form a subgroup.

On “tall” trees (where sampling is reliable), species from Central and South America form the *P. tandilensis* (Pilg.) Rahn + *P. brasiliensis* Sims + *P. bismarckii* Nederl. clade, *P. grandiflora* Meyen clade, *P. sericea* Ruiz & Pav. grade (including *P. lamprophylla* Pilg., *P. nivea*, *P. helleri*, *P. linearis* Kunth, and *P. tolucensis* Pilg.) and ser. *Hispiduleae* clade. The latter also includes *P. johnstonii* Pilg. and samples of *P. litorea* Phil. collected in Peru (thus extending the range of this Chilean species). Samples of some *P. sericea* subspecies do not branch together with the bulk of *P. sericea* samples (Figure 5D).

The “Mediterranean clade” corresponds with sects. Montana (*Plantago atrata* Hoppe, *P. monosperma* Pourr., *P. cafra* Decne., *P. nivalis* Boiss.), Lancifolia (*P. altissima* L., *P. argentea* Chaix, *P. lanceolata* L., *P. loeflingii* L., P lagopus L.), and Albicans (except *P. ciliata*). The first subclade formed with members of the first two sections plus *P. lagocephala* Bunge and two species from the *Plantago* sect. *Albicans* ser. *Minutae*: *P. minuta* Pall. and *P. lachnantha* Bunge. Sections Montana and Lancifolia are represented as proposed by Rahn [2] except for *P. loeflingii* (it groups with *Plantago* sect. *Lancifolia* instead of *Plantago* sect. *Montana*).

The second subclade of the “Mediterranean clade” consists mostly of species from *Plantago* sect. *Albicans*. *Plantago amplexicaulis* Cav. (*Plantago* sect. *Bauphula*) and *P. stocksii* Boiss. ex Decne. (*Plantago* sect. *Albicans* ser. *Ciliatae*) group together on the base of this group. The next branch(es) is *P. ovata* Forssk. and *P. psammophila* Agnew & Chal.-Kabi + Ethiopian *P. annua*. The rest of this subclade consists of species from ser. *Albicantes* and Ciliatae, plus *P. notata* Lag.

### 3.8. Morphological and Combined Analyses

**Figure 6:**
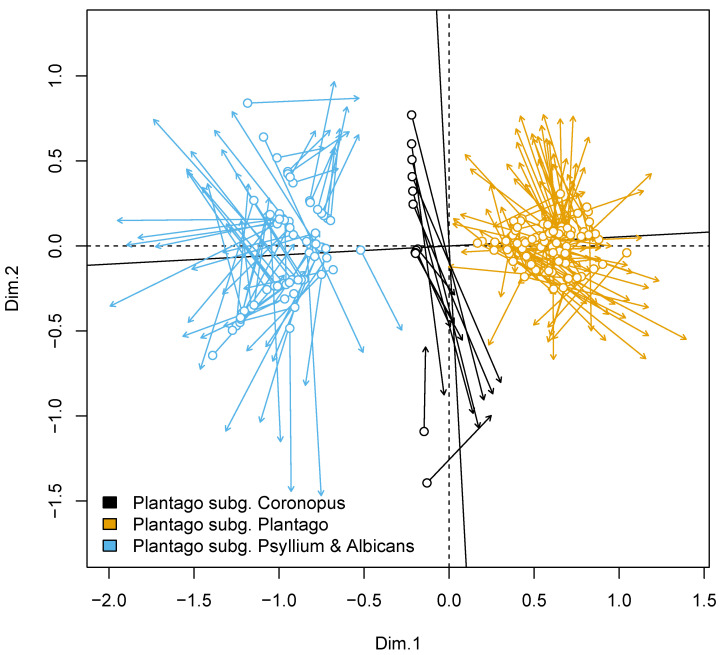
Scatterplot of the data points from joint molecular and morphological datasets (genus *Plantago* only) after the Procrustes superimposition. Differences in location of each species designated with arrows. These arrows start from the location defined by molecular data and target to the location defined by morphological data. The angle between solid and dashed axes reflects the overall Procrustean distance.

Procrustes analysis allows for the embedding of two multivariate datasets ([56,57]) and related statistical tests. Our molecular and morphological datasets are significantly correlated (Procrustes correlation = 0.7748, with significance = 0.001 based on 999 permutations), but individual placements are variably shifted (Figure 6).

**Figure 7:**
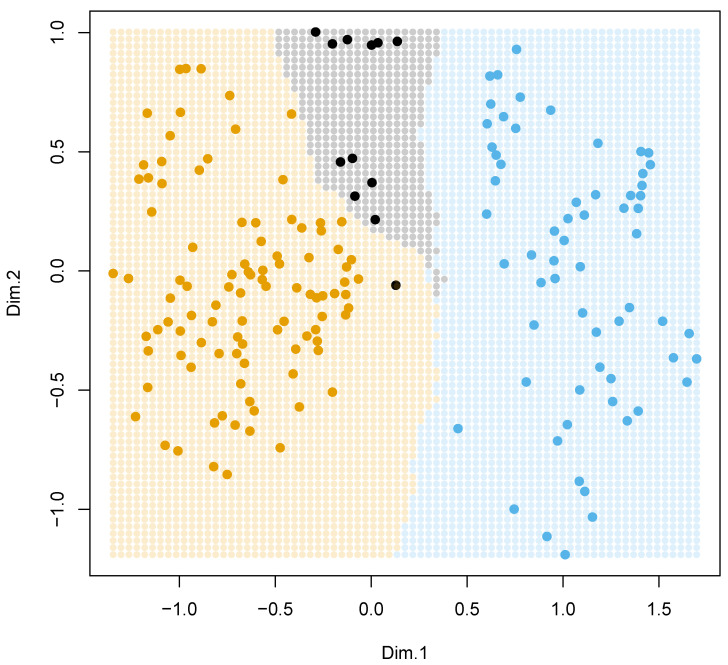
*k*-nearest neighbors (*k*-NN) plane predicts *Plantago* subgenera from the combined molecular and morphological data. The combined data was projected into a 2-dimensional plane. Solid dots correspond to *Plantago* subgg. *Plantago* (brown), *Coronopus* (black), *Psyllium* and *Albicans* (blue). For each location shown as a semi-transparent dot, subgeneric placement learned with the k-NN algorithm, and the dot was colored following this prediction. Now, if any new species appear which corresponds with any of these semi-transparent dots, its subgeneric placement is already predicted.

Even after intensive sampling, there are species which still lack the molecular information. With *k*-nearest neighbor machine learning (Figure 7), we obtained the section placements of these species. More than half of them placed with relatively high (>90 BPP) confidence (Table S4).

**Figure 8:**
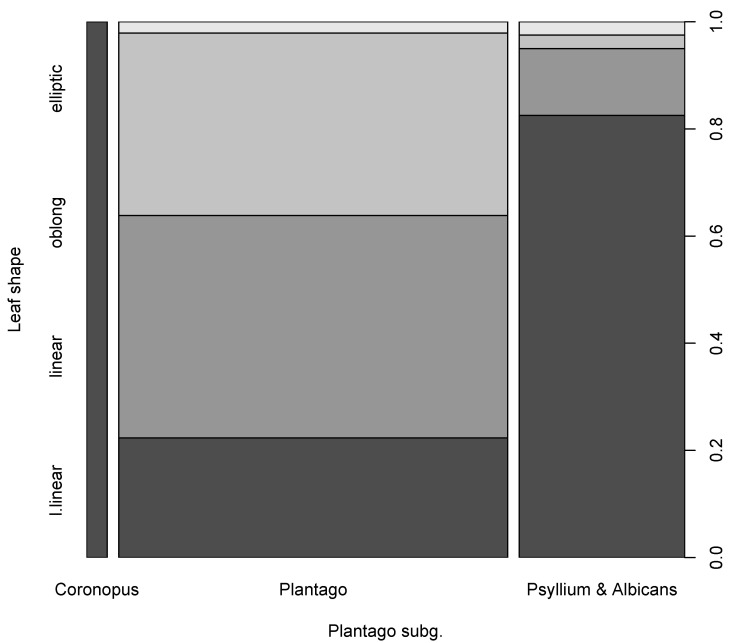
*Plantago* leaf shapes vs. *Plantago* subgenera. This plot derived from the cross-tabulation which uses a morphometric dataset for leaf shapes, and shows the proportion of different leaf shapes across three subgenera. Bar widths are proportional to the number of species in each subgenus. The shades of gray separate different character states.

Chi-squared tests returned significant *p*-values (0.0005, 0.016, and 0.0055 respectively) and typically large effect sizes (corrected Cramér’s V 0.39, 0.34 and 0.26 respectively, see also Figure 8) when comparing leaf shapes (morphometric dataset) by subgenera, sections, and macro-regions. The presence of a gapped spike was significantly different in comparisons between sections (p-value 0.0004 and 0.48 corrected Cramér’s V). At the same time, the relative sizes of the stalk and spike were not significant in all comparisons.

**Figure 9:**
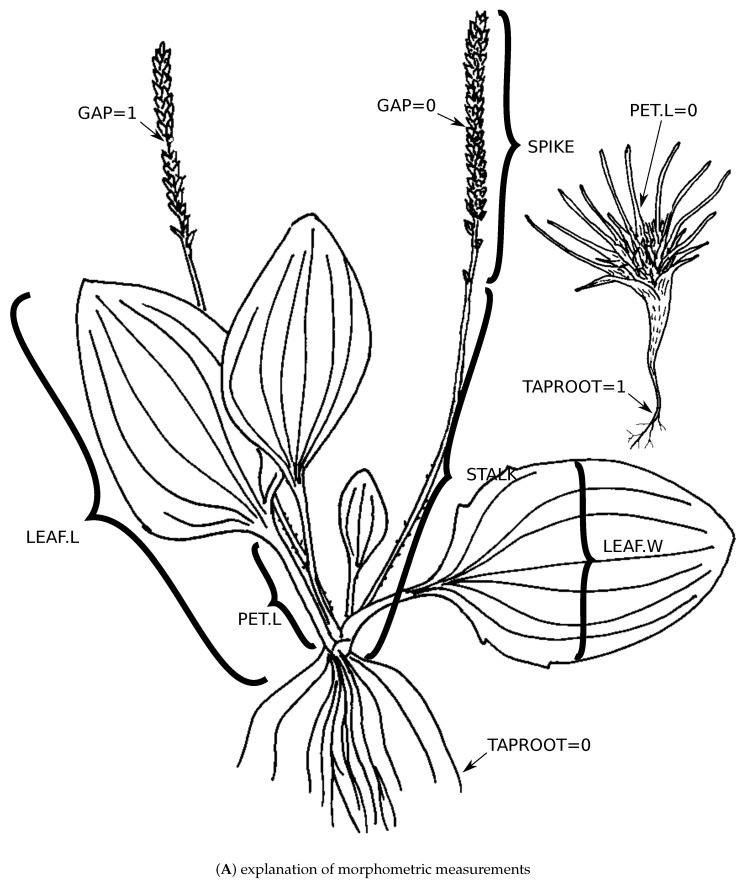

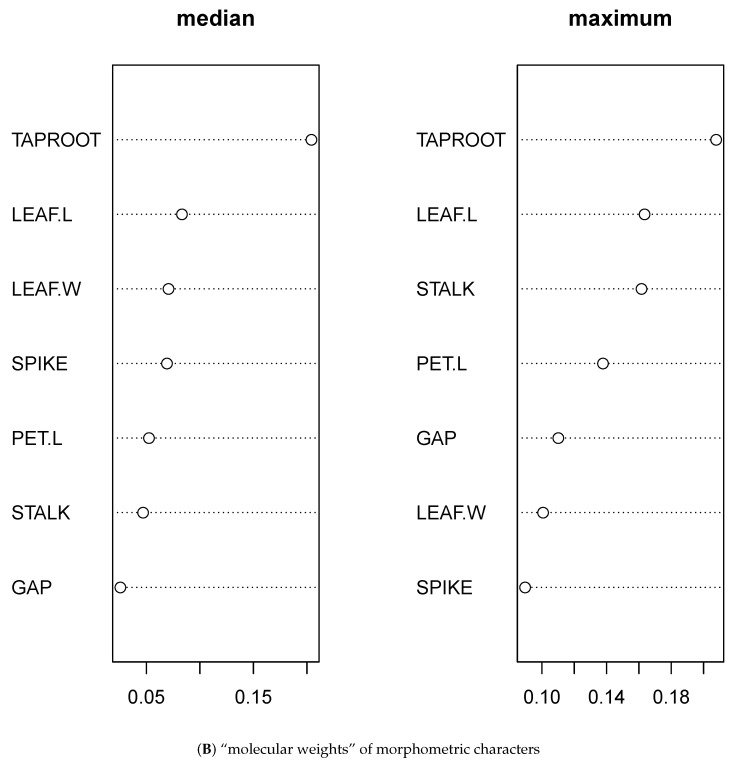

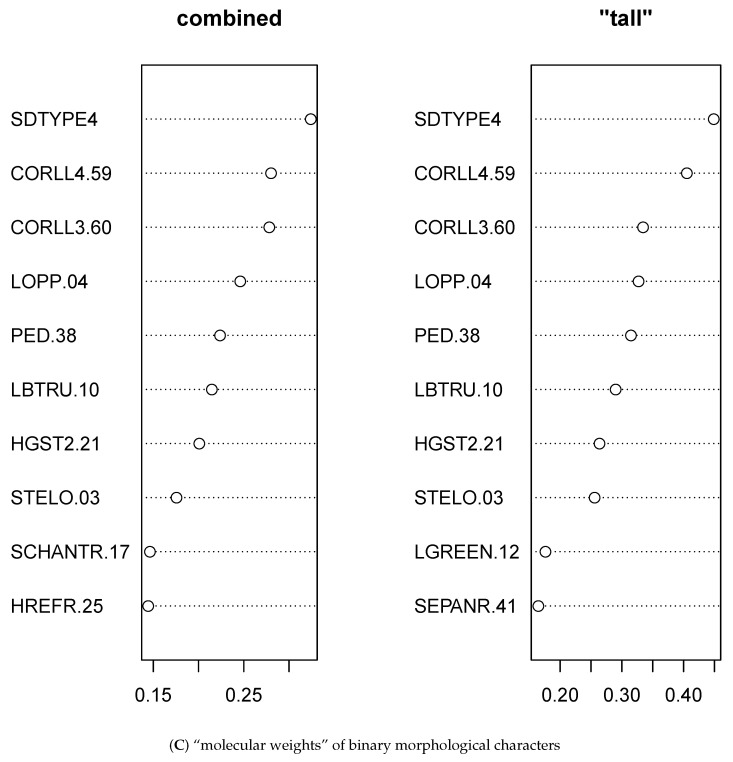
Morphological characters in *Plantago*: (**A**) explanation of morphometric measurements, character abbreviations are: PET.L petiole, LEAF.L leaf, SPIKE spike, and STALK scape lengths, LEAF.W maximal leaf width, TAPROOT presence of taproot, and GAP presence of gaps in the inflorescence; (**B**) “molecular weights” (median and maximum average Spearman correlations on 1000 bootstrap replicates) of morphometric characters, character abbreviations are the same; (**C**) “molecular weights” (average correlations between tree data and combined data) of binary characters, character abbreviations are explained in the Table S3. White dots are data values.

Among morphometric characters (Figure 9A), most significant in respect to the molecular phylogenetic tree, was the presence of a taproot, and then the length of leaves (Figure 9B). The top 10 binary morphological characters (Figure 9C) were: seed surface type 4 (with elongated ridges: [67]), long corolla (> 3 mm) lobes, opposite leaves, presence of pedicel, truncated base of leaf blade, presence of glandular hairs, elongated stem, antrorse hairs on the stalk, and presence of non-glandular hairs with the strongly refracted walls.

**Figure 10:**
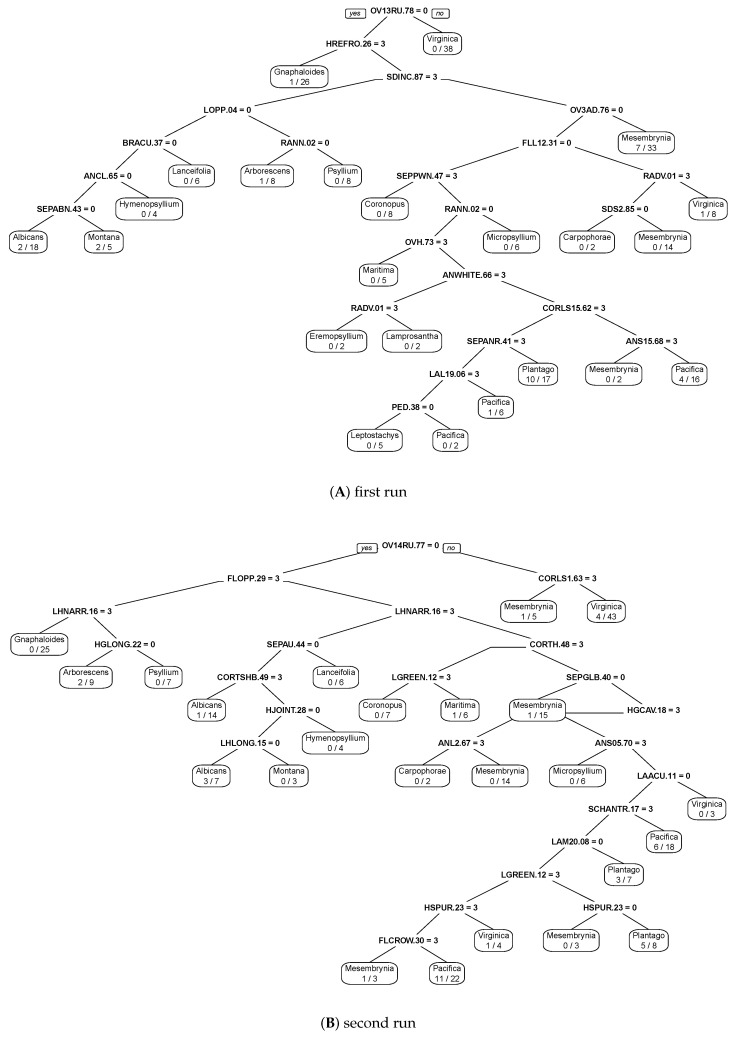
Recursive partitioning of *Plantago* sections with morphological characters, prototype of diagnostic key: (**A**) first run and (**B**) second run on binary morphological characters. Character abbreviations explained in Table S3.

We used recursive partitioning ([59]) to construct classification trees of the group (Figure 10A,B). To find as many useful characters as possible, we ran this analysis three times, excluding characters used in the previous run. The resulting recursive classification trees employed 20, 20, and 19 characters (out of 115) and had 25.3%, 35.5%, and 48.1% misclassification errors, respectively. With morphometric characters, the resulting tree used all seven characters and returned a 75.5% misclassification error.

## 4. Discussion

### 4.1. Plantagineae in General

The inclusion of the COI marker might in theory alter the outcome of our analyses because there is an evidence [68] of horizontal transfer in COI analyses of other plant groups. In case of our single-marker COI tree (Figure S1), congruence tests suggest that it is not discordant with other markers.

### 4.2. Relationships of Plantago, Aragoa and Litorella within the tribe

There is unequivocal support for the *Aragoa* (*Littorella* (*Plantago*)) structure of the group phylogeny (Figure 1, Figure 2 and Figure 4). This structure is concordant with the current understanding of the evolution of the tribe, including the evolution of flower symmetry ([8]).

Littorella, with three distinct species lineages, is robustly supported as a sister group of *Plantago*. Our data (Figure 4 and Figure 5A) agree with a view of morphologically and ecologically outstanding *Littorella* as a separate generic lineage ([21]).

### 4.3. Species Relationships within Aragoa

Bello et al. [23] included only three species (plus two hybrids) of *Aragoa* and did not resolve their relationships. Here for the first time, a significant part of the *Aragoa* diversity was reviewed using molecular data (Figure 4 and Figure 5A). In general, there is some support for groupings previously obtained based solely on morphology ([1]).

However, some morphologically unusual species like *A. dugandii* with relatively broad leaves, and *A. perez-arbelaeziana* with long tubular flowers do not make separate clades but are clustered together with more “typical” species (*A. lycopodioides* and *A. romeroi*, respectively, both from the main clade). The relatively broad, patent leaves and the simple inflorescence of *A. dugandii*, together with relatively large flowers, might be therefore interpreted as an adaptation to environments where moisture loss is not so critical as for many other species of the genus. As for *A. perez-arbelaeziana*, the presence of long, pendulous, yellowish corollas, unusual in the genus, can be interpreted as a result of recent adaptive radiation to a specific type of pollinator (hummingbirds: Fernández personal comm./observation) which does not entail the significant modifications in vegetative structures.

The machine learning placement of three unsampled *Aragoa* species resulted in the selection of possible candidate neighbors (Table S4) from the same main *Aragoa* clade.

### 4.4. Subgeneric Relationships within Plantago

In general, there are no principal conflicts with the most recent taxonomic studies of the genus based on morphology summarized in [2]. However, there are numerous differences worth emphasizing.

All our analyses reproduce the ((*Coronopus*, *Plantago*), (*Bougueria*, (*Psyllium*, *Albicans*))) backbone (Figure 4 and Figure 5B–D); this is also visible on the phylogenetic density map (Figure 3). Our results correspond well with [3,4,12]. In addition, we provide strong support for the separate *Psyllium* and *Albicans* clades, which we treat here as subgenera based on the molecular and morphological evidence.

### 4.5. Plantago subg. Plantago

In general, our trees in this part (Figure 5B,C) do not provide a clear, well-resolved picture as in plastome studies ([4]). Our results are generally well in line with previous studies but comparing with [4,12,33,69,70] we were able to obtain information about many species previously not studied molecularly, and also about species which were not included in plastome research [4]. In several cases, the position of species in the specific section was “inferred based on the accumulated knowledge.” Many such species are now assigned to sections (Table S4), and this is reflected in our working classification of Plantagineae (Table S2).

For example, it is mentioned in [4] that “… based on our phylogeny it is impossible to infer the position of the five unsampled American species … *P. barbata*, *P. correae*, *P. pulvinata*, *P. sempervivoides*, and *P. uniglumis* …”. Our current trees (Figure 5C) place these species (except *P. correae*, for which we have no data) in one clade, which also includes *P. rigida*, *P. tubulosa*, *P. moorei*, *P. tehuelcha*, and *P. fernandezia*, the placement of which is well justified geographically (Table S5). Morphologically unusual *P. sempervivoides* is sister to the remainder of the group. Two *Plantago* sect. *Carpophorae* species are sister to *P. fernandezia* + *P. barbata*. This group likely has an Andean origin, and *P. fernandezia* might, therefore, have arrived at Juan Fernández from South America ([12,71]). The positions of *P. fernandezia*, *P. tehuelcha*, and *Plantago* sect. *Carpophorae* species are different on the plastome trees ([4]), but these trees have low support exactly in these parts.

Another complicated group was not resolved entirely, but we provide several insights for the placements and phylogeny in general of species around polymorphic and widespread *P. asiatica* ([69,70,72]). Most of these forms cluster together (Figure 4 and Figure 5C) and separately from the *P. major* clade; thus, morphological similarity here does not justify taxonomic closeness. Japanese endemic *Plantago hakusanensis* is either within this group or sister to the rest of this group; the same is true for *P. hasskarlii*. Relations of *P. hakusanensis* and *P. hasskarlii* mandate more in-depth research.

Molecular data from *Plantago japonica*, together with ecology and morphology, suggests that this species is likely distinct from *P. major* ([70,72]).

Samples from the Hubei province of China (“*Plantago* sp. Hupeh1”) appear similar to *Plantago asiatica* but branch near *P. komarovii* + *P. camtschatica* clade (Figure 5B). As the allopolyploid origin of tetra- or hexaploid *P. asiatica* and *P. rugelii* was suggested in [70], we cannot exclude the possibility of the allopolyploid origin of these Hubei samples (with ITS kept from the *Plantago* sect. *Pacifica* parent). We believe that a thorough study of Chinese, Korean, and Japanese *Plantago* subg. *Plantago* species (and specifically species from sect. Pacifica) is required before reaching any robust conclusions. In the light of [70], the recent historical origin of *P. rugelii* (which branches closely to *P. sparsiflora* on our trees but morphologically hardly distinguishable from *P. major*) might also be justified.

There are seven endemic *Plantago* species described from New Guinea (*P. aundensis*, *P. depauperata*, *P. montisdicksonii* P.Royen, *P. papuana* P.Royen, *P. polita*, *P. stenophylla* Merr. & L.M.Perry and *P. trichophora* Merr. & L.M.Perry), but DNA extraction from available samples failed in 90% of cases. Nevertheless, we were able to place four of these species (Figure 5B): *P. aundensis* with Hawaiian *P. pachyphylla* s.l. and *P. hawaiensis* (the third Hawaiian species, *P. princeps* does not hold a stable position on our trees); *P. depauperata* and *P. polita* with Australian *P. muelleri* Pilg.; and *P. trichophora* with Australian *P. gaudichaudii* Barnéoud. Still, much more sampling is needed, and Hawaiian species also deserve a closer look ([4]).

**Figure 11:**
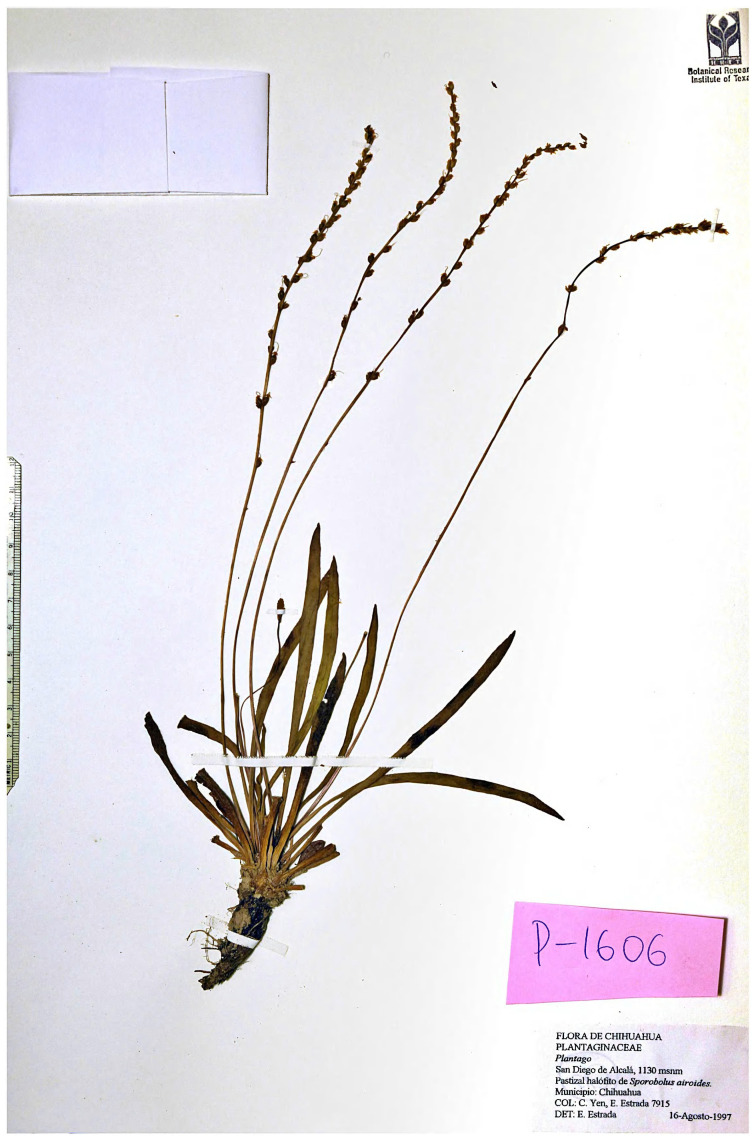
*Plantago chihuahuensis*, photograph of the herbarium sample (holotype, BRIT).

Morphologically unusual samples (Figure 11) from Chihuahua (Mexico) are superficially similar to *P. gaudichaudii* from Australia and *P. remota* from South Africa, but we believe that this is a clear example of morphological convergence. On our trees, these samples belong to *Plantago* sect. *Pacifica* clade and branch together with Midwestern *P. eriopoda* and *P. tweedyi* from the Rocky Mountains region (Figure 5B). We believe that these samples deserve to be described as a new species of *Plantago* (see below).

The same Pacifica clade includes on “broad” trees *Plantago macrocarpa*, the Northern Pacific seashore species. On the “tall” trees (Figure 5B), the placement of this species is not stable.

*Plantago krascheninnikovii* is superficially similar to the inland forms of *P. maritima* and therefore treated as a member of *Plantago* subg. *Coronopus* ([73]). Known populations of this rare Urals species do not typically form ripe seeds ([53]), thus preventing the correct placement on the base of morphology. Here we confirm for the first time its similarity with other Lamprosantha species, for example, Eastern European *P. schwartzenbergiana* (Figure 5C).

The southern tetraploid forms similar to *Plantago media* but with thick, erect, grayish leaves ([53,73]), often regarded as *P. urvillei* Opiz or *P. media* subsp. stepposa (Kuprian.) Soó are distant from *P. media* s.str. (Figure 5C), thus necessitating the separation of this taxon. However, the diversity of *P. media* s.l. is still far from being fully understood ([74]), and more research is needed to draw robust taxonomic conclusions. Here should be noted that *P. media* subsp. stepposa must not be mixed with the similarly looking shade, mesophytic plants of *P. media* ([53]).

Plants from Öland described as *P. minor* Fr. are morphologically distinct and geographically isolated from other species of this section. However, common garden experiments ([75]) led to the conclusion that they are conspecific with *P. tenuiflora*. On our trees, the sample from Öland is proximal to *P. polysperma*. We believe that more research on Öland plantains is needed to understand the taxonomic status of these forms better.

Andean *Plantago oreades* Decne. with distinct morphology (narrow leaves, long inflorescences, broad bracts, 1–3 seeded fruit, thick roots) was nevertheless included into broadly understood *P. australis* ([2]). On our trees (Figure 5C), it almost always separate from the other *P. australis* samples. Therefore, we propose here to re-establish this species.

### 4.6. Plantago subg. Coronopus

Our trees (Figure 4 and Figure 5B) provide one of the most comprehensive phylogenies for the *Plantago* subg. *Coronopus*, and are in concordance with the recent work of [32]. In addition, we were able to place several species which have not been the subject of molecular studies before. The most interesting are positions of Canarian *P. asphodeloides* Svent as sister to the rest of species from *Plantago* sect. *Coronopus*, and *P. eocoronopus* Pilg. as sister to the rest of the *Plantago* sect. *Maritima*. The latter species is the rare Afghan plant, practically absent in collections. Pilger ([30]) guessed this species to be ancestral for the section, and now we can support this view on the basis of both molecular markers and morphology ([61]). All our “*P. schrenkii*” C. Koch samples from the Arctic are identical to *P. maritima* ([76]).

### 4.7. Plantago subgg. Bougueria, Albicans and Psyllium

Generally, this part of our trees (Figure 4 and Figure 5D) is the most congruent with the classification [2]. Our data is also congruent with the most complete (until now) sampling of the group ([3]) and provides robust support for many sub-groupings, which is reflected in our working classification (Table S2).

Among other results, we found that likely extinct *P. johnstonii* ([14]) branches close to the coastal annual *P. limensis* and therefore belongs not to ser. *Brasilienses* but to ser. *Hispiduleae*. It is possible then that the perennial life form of the former species is the result of adaptation to the “lomas” microclimate.

The most recent review of the *Plantago* sect. *Lancifolia* ([77]) is in agreement with our results but also provides new insights for the classification of this Mediterranean taxon. More research is needed to understand the relations of rare endemic species in this group.

### 4.8. Integration of Molecular and Morphological Data

With Procrustes analysis (Figure 6), we found that the overall “picture of diversity” is retained between morphological and molecular approaches. In other words, the correspondence between these analyses is high. This in turn allows for the *k*-nearest neighbor (Figure 7) placement of several species (Table S4) which might be otherwise *incertae sedis* in our working classification (Table S2).

Within sections and subgenera, we found several repetitive morphometric patterns (Figure 8) which are present in each taxonomic group of particular level (“refrains”: [78]); this is additional evidence that morphometric characters should work better within sections ([32]). We also found several morphological and morphometric characters most correlated with molecular phylogenies (Figure 9). The morphometric characters are especially interesting because the analysis was performed on the same samples and not on higher units like species descriptions. Notable is the importance of the presence of taproot, which is another argument for collecting plantains always with carefully preserved underground parts. Among the binary morphological characters, attention should be paid on the research of seed surface characters ([67]), highly correlated with molecular data (Figure 9C).

### 4.9. Identification Keys

Production of identification keys is a complicated task in plantains. These keys must take into account the high variability and overlapping of the most distinctive characters used in *Plantago* taxonomy ([4]). Therefore, it might be desirable to employ here results of machine learning techniques such as recursive partitioning. Partitioning trees (Figure 10A,B) are able to identify the most informative characters and distinguish groups with minimal errors. We used these partitioning trees as a basis of the prototypic dichotomous key to *Plantago* sections. This prototypic key might serve as a framework for the future development of even more comprehensive keys:

1a. Ovary with 1–3 ovules and a rudiment of an upper compartment on the adaxial side of the placenta. Corolla lobes longer than 1 mm. Flowers are mostly cleistogamous; corolla lobes form a beak . . . . . . . . . . . . . . . . *Plantago* sect. *Virginica*
1b. Ovary structured otherwise. Corolla lobes short or long. Flowers are mostly chasmogamous, corolla in most (but not all) species does not form a beak . . . . . . . . . . . . . . . . . . . . . . . . . . . . . . . . . . . . . . . . . . . . . . . . . . . . . . . . . . . . . . . . . . . . . . . . . . . . 2.
2a. Non-glandular hairs with joints are strongly refracting, walls between cells oblique. Hairs on leaves narrow, less than 0.04 mm . . . . . . . . . . . . . . . . . . . . . . . . . . . . . . . . . . . . . . . . . . . . . . . . . . . . . . . . . . . . . . . . . . . . . . . . . *Plantago* sect. *Gnaphaloides*
2b. Strongly refracting joints absent. Hairs on leaves (if present) variable . . . . . . . . . . . . . . . . . . . . . . . . . . . . . . . . . . . . . . . . . . 3.
3a. The inner side of the seed is deeply concave . . . . . . . . . . . . . . . . . . . . . . . . . . . . . . . . . . . . . . . . . . . . . . . . . . . . . . . . . . . . . . . 13.
3b. The inner side of the seed is not deeply concave . . . . . . . . . . . . . . . . . . . . . . . . . . . . . . . . . . . . . . . . . . . . . . . . . . . . . . . . . . . . 4.
4a. Ovary with a third compartment at the top on the adaxial side of the placenta, or with a rudiment of it, seen as a thickening at the apex on the posterior side of the ripe placenta. If this compartment absent, then there are few flowers in the inflorescence; no adventitious roots and seeds are longer than 2 mm. Sepals are glabrous on the back . . . . . . . . . . . . . . . . . . . . . . . . . . . . . . . . . . . . . . . . . . . . . . . . . . . . . . . . . . . . . . . . . . . . . . . . . . . . . . . . . . . . . . . . . . . . *Plantago* sect. *Mesembrynia*
4b. Ovary without the third compartment, other character combinations are different . . . . . . . . . . . . . . . . . . . . . . . . . . . . . . . 5.
5a. Less than four flowers per inflorescence. Carpophore present . . . . . . . . . . . . . . . . . . . . . . . . . . . *Plantago* sect. *Carpophorae*
5b. Inflorescence with more than 12 flowers. Carpophore absent . . . . . . . . . . . . . . . . . . . . . . . . . . . . . . . . . . . . . . . . . . . . . . . . . 6.
6a. Posterior sepals with the membranaceous, very conspicuous wing on the back. Leaves are usually remaining green on drying. Corolla tube hairy. Annuals; leaves are often dentate or even dissected . . . . . . . . . . . . . . . *Plantago* sect. *Coronopus*
6b. Posterior sepals without a conspicuous wing on the back. Leaves dry differently. Corolla tube hairy or glabrous. Annuals or perennials; leaves with the whole margin or sometimes dentate . . . . . . . . . . . . . . . . . . . . . . . . . . . . . . . . . . . . . 7.
7a. Annuals. Anthers usually less than 0.5 mm long . . . . . . . . . . . . . . . . . . . . . . . . . . . . . . . . . . . . . *Plantago* sect. *Micropsyllium*
7b. Perennials. Anthers are longer than 0.5 mm . . . . . . . . . . . . . . . . . . . . . . . . . . . . . . . . . . . . . . . . . . . . . . . . . . . . . . . . . . . . . . . . 8.
8a. Ovary hairy. Corolla tube hairy. Leaves usually do not remain green on drying . . . . . . . . . . . . . . . . *Plantago* sect. *Maritima*
8b. Ovary glabrous. Corolla tube glabrous. Leaves usually remain green on drying . . . . . . . . . . . . . . . . . . . . . . . . . . . . . . . . . 9.
9a. Anthers white both when fresh and dried . . . . . . . . . . . . . . . . . . . .. . . . . . . . . . . . . . . . . . . . . . . . . . . . . . . . . . . . . . . . . . . . . 10.
9b. Anthers not white . . . . . . . . . . . . . . . . . . . . . . . . . . . . . . . . . . . . . . . . . . . . . . . . . . . . . . . . . . . . . . . . . . . . . . . . . . . . . . . . . . . . . 11.
10a. Root system mostly of primary and secondary roots . . . . . . . . . . . . . . . . . . . . . . . . . . . . . . . . . . *Plantago* sect. *Lamprosantha*
10b. Root system mostly of adventitious roots . . . . . . . . . . . . . . . . . . . . . . . . . . . . . . . . . . . . . . . . . . . *Plantago* sect. *Eremopsyllium*
11a. Corolla lobes longer than 1.5 mm. Ovary with four or fewer ovules. Leaf width usually less than 25 mm . . . . . . . . . . . . . . . . . . . . . . . . . . . . . . . . . . . . . . . . . . . . . . . . . . . . . . . . . . . . . . . . . . . . . . . . . . . . . . . . . . . . . . . . . . . . . . . . . *Plantago* sect. *Pacifica*
11b. Corolla lobes shorter than 1.5 mm. Ovary usually with four or more ovules. Leaf width more than 25 mm . . . . . . . . . 12.
12a. Anterior sepals distinctly narrower than posterior, and differently shaped . . . . . . . . . . . . . . . . *Plantago* sect. *Leptostachys*
12b. Anterior and posterior sepals similar . . . . . . . . . . . . . . . . . . . . . . . . . . . . . . . . . . . . . . . . . . . . . . . . . . . *Plantago* sect. *Plantago*
13a. Leaves opposite or in whorls of three . . . . . . . . . . . . . . . . . . . . . . . . .. . . . . . . . . . . . . . . . . . . . . . . . . . . . . . . . . . . . . . . . . . . 14.
13b. Leaves alternate . . . . . . . . . . . . . . . . . . . . . . . . . . . . . . . . . . . . . . . . . . . . . . . . . . . . . . . . . . . . . . . . . . . . . . . . . . . . . . . . . . . . . 15.
14a. Perennials, typically without long glandular hairs. Inner bracts narrow. Seeds longer than 3 mm . . . . . . . . . . . . . . . . . . . . . . . . . . . . . . . . . . . . . . . . . . . . . . . . . . . . . . . . . . . . . . . . . . . . . . . . . . . . . . . . . . . . . . . . . . . . . . . . . . . . . *Plantago* sect. *Arborescens*
14b. Annuals, with long glandular hairs. Inner bracts are broad. Seeds shorter than 3 mm . . . . . . . . . . *Plantago* sect. *Psyllium*
15a. Bract with the upper part scarious, acuminate. Some species with anterior sepals united for more than half of their length . . . . . . . . . . . . . . . . . . . . . . . . . . . . . . . . . . . . . . . . . . . . . . . . . . . . . . . . . . . . . . . . . . . . . . . . . . . . *Plantago* sect. *Lancifolia*
15b. Bract without scarious, acuminate upper part. Anterior sepals always free . . . . . . . . . . . . . . . . . . . . . . . . . . . . . . . . . . . . 16.
16a. Connective of anther very large, about as long as the pollen sacs. Plants densely hairy (leaf surface hardly visible), cells of non-glandular hairs jointed by a common wall with crown-like elongations . . . . . . . . . *Plantago* sect. *Hymenopsyllium*
16b. Connective of anther smaller. Plants are not densely hairy, cells of hairs without crown-shape elongations . . . . . . .. 17.
17a. The nerve of anterior sepals well developed. Corolla lobes are slightly hairy on the back. The concave inner side of the seed covered by a ragged, white membrane, except for two areas to the right and left of the center. Leaves are usually remaining green on drying . . . . . . . . . . . . . . . . . . . . . . . . . . . . . . . . . . . . . . . . . . . . . . . . . . . . . . . . . . . . *Plantago* sect. *Albicans*
17b. The nerve of anterior sepals present at base only, distal part scarious. Corolla lobes not hairy. White membrane on seeds absent. Leaves usually darken on drying . . . . . . . . . . . . . . . . . . . . . . . . . . . . . . . . . . . . . . . . . . . *Plantago* sect. *Montana*

### 4.10. Description of the New Species

*Plantago chihuahuensis* Shipunov, sp.nov.

Differs from *Plantago eriopoda* and *P. tweedyi* by narrow and long leaves (0.4–0.6 cm × 9–15 cm), tall inflorescences (30–35 cm), and sparsely (with gaps 0.5–1 cm in the middle of spike) arranged flowers. From *P. sparsiflora*, it differs by ecology (semi-deserts, on alkaline soils), narrow leaves, and shorter inflorescences.

TYPE:— San Diego de Alcalá, 1130 msnm. Pastizal halóhito de *Sporobolus airoides*. Municipio: Chihuahua, 16 August 1997, C. Yen & E. Estrada 7915 (holotype: BRIT!).

Etymology:—Named after the region of collection, Chihuahua state of Mexico.

Distribution:—MEXICO, CHIHUAHUA. (BRIT C. Yen, E. Estrada 5644!).

Description:—Plants perennial; roots taproots, thick. Stems about 1 cm. Leaves 0.4–0.6 cm × 9–15 cm; blade linear, margins entire, veins inconspicuous, surfaces glabrous. Scapes 30–35 cm, spikes brownish, 70–140 cm, very loosely flowered (rachis visible between flowers); bracts ovate, 1.5–2 mm, length 0.6–0.8 times sepals. Flowers: sepals 2 mm; corolla radially symmetric, lobes are spreading, 1 mm; stamens 4.

Notes:—Very little is known about habitat, ecology and phenology of this species. Collected specimens grow on alkaline soils, in grass semi-desert on the relatively high altitudes (about 1 km above sea level). Flowering time is likely the second half of summer (August). As only two specimens are known, more observations and collections are needed. This species is definitely rare and must be protected, and we hope that describing this new species, we will improve its chances to survive the Anthropocene.

## Data Availability

Not applicable.

## Supplementary Materials

The following are available online https://www.mdpi.com/article/10.3390/plants10112299/s1, Table S1: Vouchers, Table S2: Working classification of Plantagineae, Table S3: Morphological characters, Table S4: Machine learned placements of the molecularly under-sampled species of *Plantago*, Table S5: Geographical origin of *Plantago* species, Figure S1: The single-marker *Plantago* phylogenetic tree based on COI sequences; this tree is similar to other trees suggesting (together with CADM tests) the absence of HGT.

## References

[1] Fernández A.J.L. (1995). Scrophulariaceae–Aragoeae. Flora de Colombia.

[2] Rahn K. (1996). A phylogenetic study of the Plantaginaceae. Bot. J. Linn. Soc..

[3] Rønsted N.A.H., Chase M.W., Albach D.C., Bello M.A. (2002). Phylogenetic relationships within *Plantago* (Plantaginaceae): Evidence from nuclear ribosomal ITS and plastid *trn*L-F sequence data. Bot. J. Linn. Soc..

[4] Hassemer G., Bruun-Lund S., Shipunov A., Briggs B.G., Meudt H.M., Rønsted N.A.H. (2019). The application of high- throughput sequencing for taxonomy: The case of *Plantago* subg. *Plantago* (Plantaginaceae). Mol. Phylogenet. Evol..

[5] Hassemer G., De Giovanni R., Trevisan R. (2016). The use of potential distribution models in the study of the distribution and conservation status of plants: The case of *Plantago* L. (Plantaginaceae) in *Brazil*. J. Torrey Bot. Soc..

[6] Linnaeus C. (1754). Genera Plantarum.

[7] Linnaeus C. (1753). Species Plantarum.

[8] Preston J.C., Ciera C.M., Hileman L.C. (2011). Gradual disintegration of the floral symmetry gene network is implicated in the evolution of a wind-pollination syndrome. Proc. Natl. Acad. Sci. USA.

[9] Cho Y., Mower J.P., Qiu Y.L., Palmer J.D. (2004). Mitochondrial substitution rates are extraordinarily elevated and variable in a genus of flowering plants. Proc. Natl. Acad. Sci. USA.

[10] Asaf S., Khan A.L., Lubna A.K., Khan A., Khan G., Lee I.J., Al-Harrasi A. (2020). Expanded inverted repeat region with large scale inversion in the first complete plastid genome sequence of *Plantago ovata*. Sci. Rep..

[11] Carlquist S. (1970). Wood anatomy of insular species of *Plantago* and the problem of raylessness. Bull. Torrey Bot. Club.

[12] Iwanycki Ahlstrand N.E., Verstraete B., Hassemer G., Dunbar-Co S., Hoggard R., Meudt H.M., Rønsted N.A.H. (2019). Ancestral range reconstruction of remote oceanic island species of *Plantago* (Plantaginaceae) reveals differing scales and modes of dispersal. J. Biogeogr..

[13] Hassemer G., Rønsted N.A.H. (2016). Yet another new species from one of the best-studied neotropical areas: *Plantago humboldtiana* (Plantaginaceae), an extremely narrow endemic new species from a waterfall in southern Brazil. PeerJ.

[14] Hassemer G., Shipunov A., Rønsted N.A.H., Meudt H.M. (2018). Taxonomic and geographic novelties in the genus *Plantago* (Plantaginaceae) in Chile, including the description of a new species. Phytotaxa.

[15] Hassemer G. (2019). Novelties and notes on *Plantago* sect. *Virginica* (Plantaginaceae), including the description of a new species and a revised identification key. Webbia.

[16] Di Pietro R., Iamonico D., Soldano A. (2013). Proposal to conserve the name *Plantago serpentina* against *P. strictissima* (Plantaginaceae). Taxon.

[17] Doweld A.B., Shipunov A. (2017). Proposal to reject the name *Plantago indica* (Plantaginaceae). Taxon.

[18] Hassemer G. (2018). Notes on the montane Indo-Iranian species in *Plantago* subgenus *Plantago* (Plantaginaceae). Phytotaxa.

[19] Hassemer G. (2018). Advances to the taxonomic knowledge of *Plantago subulata* (*Plantago* sect. *Maritima*, Plantaginaceae). Turk. J. Bot..

[20] Bergius P.J. (1768). *Littorella juncea*, en svensk växt. Kongliga Vetensk. Acad. Handl..

[21] Hoggard R.K., Kores P.J., Molvray M., Hoggard G.D., Broughton D.A. (2003). Molecular systematics and biogeography of the amphibious genus *Littorella* (Plantaginaceae). Am. J. Bot..

[22] Kunth K.S. (1818). Nova Genera et Species Plantarum.

[23] Bello M.A., Chase M.W., Olmstead R.G., Rønsted N.A.H., Albach D. (2002). The páramo endemic *Aragoa* is the sister genus of *Plantago* (Plantaginaceae; Lamiales): Evidence from plastid *rbc*L and nuclear ribosomal ITS sequence data. Kew Bull..

[24] Fernández A.J.L. (1993). Novedades taxonómicas en *Aragoa* H.B.K. (Scrophulariaceae) y sinopsis del género. An. Jardín Botánico Madr..

[25] Fernández A.J.L. Algunos patrones de distribución y endemismo en plantas vasculares de los páramos de Colombia. Proceedings of the Memorias Congreso Mundial de Páramos.

[26] Bello M.A., Rudall P.J., González F., Fernández A.J.L. (2004). Floral morphology and development in *Aragoa* (Plantaginaceae) and related members of the order Lamiales. Int. J. Plant Sci..

[27] Dumortier B.C. (1829). Analyse des Familles des Plantes: Avec L’indication des Principaux Genres qui s’y Rattachent.

[28] Barnéoud F.M. (1845). Monographie Générale de la Famille des Plantaginées.

[29] Decaisne J. (1852). Plantaginaceae. Candolle A. Prodromus Syst. Nat. Regni Veg..

[30] Pilger R., Engler A. (1937). Plantaginaceae. Das Pflanzenreich.

[31] Mower J.P., Guo W., Partha R., Fan W., Levsen N., Wolff K., Nugent J.M., Pabón-Mora N., González F. (2021). Plastomes from tribe Plantagineae (Plantaginaceae) reveal infrageneric structural synapormorphies and localized 2 hypermutation for *Plantago* and functional loss of *ndh* genes from *Littorella*. Mol. Phylogenet. Evol..

[32] Höpke J., Mucina L., Albach D.C. (2019). Phylogenetic and morphometric analysis of *Plantago* section *Coronopus* (Plantaginaceae). Taxon.

[33] Tay M.L., Meudt H.M., Garnock-Jones P.J., Ritchie P.A. (2010). DNA sequences from three genomes reveal multiple long-distance dispersals and non-monophyly of sections in Australasian *Plantago* (Plantaginaceae). Aust. Syst. Bot..

[34] Meudt H.M. (2011). Amplified fragment length polymorphism data reveal a history of auto-and allopolyploidy in New Zealand endemic species of *Plantago* (Plantaginaceae): New perspectives on a taxonomically challenging group. Int. J. Plant Sci..

[35] Meudt H.M. (2012). A taxonomic revision of native New Zealand *Plantago* (Plantaginaceae). N. Z. J. Bot..

[36] Choi J., Lee H.J., Shipunov A. (2015). All that is gold does not glitter? Age, taxonomy, and ancient plant DNA quality. PeerJ.

[37] Funk V.A., Edwards R., Sterling K. (2018). The problem with (out) vouchers. Taxon.

[38] Seifert K.A., Crous P.W., Frisvad J.C. (2008). Correcting the impact factors of taxonomic journals by appropriate citation of taxonomy (ACT). Persoonia.

[39] Reveal J.L. Indices Nominum Supragenericorum Plantarum Vascularium. Alphabetical Listing by Genera of Validly Published Suprageneric Names. http://www.plantsystematics.org/reveal/pbio/fam/allspgnames.html.

[40] Kuzmina M., Ivanova N. Primer Sets for Plants and Fungi. http://ccdb.ca/site/wp-content/uploads/2016/09/CCDB_PrimerSets-Plants.pdf.

[41] Samarakoon T., Wang S.Y., Alford M.H. (2013). Enhancing PCR amplification of DNA from recalcitrant plant specimens using a trehalose-based additive. Appl. Plant Sci..

[42] R Core Team (2019). R: A Language and Environment for Statistical Computing.

[43] Larsson A. (2014). AliView: A fast and lightweight alignment viewer and editor for large data sets. Bioinformatics.

[44] Edgar R.C. (2004). MUSCLE: Multiple sequence alignment with high accuracy and high throughput. Nucleic Acids Res..

[45] Paradis E., Claude J., Strimmer K. (2004). APE: Analyses of phylogenetics and evolution in R language. Bioinformatics.

[46] Ronquist F., Huelsenbeck J.P. (2003). MrBayes 3: Bayesian phylogenetic inference under mixed models. Bioinformatics.

[47] Heibl C. IPS: R Language Tree Plotting Tools and Interfaces to Diverse Phylogenetic Software Packages. https://github.com/heibl/ips.

[48] Shipunov A. Shipunov: Miscellaneous Functions from Alexey Shipunov. R Package Version 1.2. https://CRAN.R-project.org/package=shipunov.

[49] Schliep K.P. (2011). Phangorn: Phylogenetic analysis in R. Bioinformatics.

[50] Stamatakis A. (2014). RAxML version 8: A tool for phylogenetic analysis and post-analysis of large phylogenies. Bioinformatics.

[51] Nixon K. (1999). The parsimony ratchet, a new method for rapid parsimony analysis. Cladistics.

[52] Campbell V., Legendre P., Lapointe F.J. (2011). The performance of the Congruence Among Distance Matrices (CADM) test in phylogenetic analysis. BMC Evol. Biol..

[53] Shipunov A. (1998). Plantains (Genera *Plantago* L. and *Psyllium* Mill.) from European Russia and Adjacent Territories. Ph.D. Thesis.

[54] Shehata A.A., Loutfy M.H.A. (2006). On the taxonomy of Plantaginaceae Juss. *sensu lato*: Evidence from SEM of the seed coat. Turk. J. Bot..

[55] Ashkenazy H., Sela I., Levy Karin E., Landan G., Pupko T. (2018). Multiple sequence alignment averaging improves phylogeny reconstruction. Syst. Biol..

[56] Peres-Neto P.R., Jackson D.A. (2001). How well do multivariate data sets match? The advantages of a Procrustean superimposition approach over the Mantel test. Oecologia.

[57] Balbuena J.A., Míguez-Lozano R., Blasco-Costa I. (2013). PACo: A novel Procrustes application to cophylogenetic analysis. PLoS ONE.

[58] Ripley B.D. (1996). Pattern Recognition and Neural Networks.

[59] Venables W.N., Ripley B.D. (2002). Modern Applied Statistics with S.

[60] Therneau T., Atkinson B., Ripley B.D. Rpart: Recursive Partitioning and Regression Trees. R Package Version 4.1-8. http://CRAN.R-project.org/package=rpart.

[61] Shipunov A. (2000). On the taxonomic status of *Plantago eocoronopus* Pilger and *P. arachnoidea* Schrenk var. *lorata* Liu. Bull. Mosc. Soc. Nat. Biol. Ser..

[62] Shipunov A., Naczi R.F.C., Abbott J.R. (2017). Portion of Plantaginaceae, the Plantain Family: *Littorella* and *Plantago*. New Manual of Vascular Plants of Northeastern United States and Adjacent Canada.

[63] Shipunov A., Freeman C., Rabeler R. (2019). *Plantago* and *Littorella*. Flora of North America.

[64] Li Z., Wei L., Hoggard R.K., Wu Z., Raven P.H., Hong D. (2011). Plantaginaceae. Flora of China.

[65] Morgan-Richards M., Wolff K. (1999). Genetic structure and differentiation of *Plantago major* reveals a pair of sympatric sister species. Mol. Ecol..

[66] Rahn K. (1974). *Plantago* section *Virginica*; A taxonomic revision of a group of American plantains, using experimental, taximetric and classical methods. Dan. Bot. Ark..

[67] Shipunov A. (1998). The significance of the sculpture of the surface of seeds for systematics of genera *Plantago* L. and *Psyllium* Mill. (Plantaginaceae). Bulletin of Moscow Society of Naturalists. Biol. Ser..

[68] Sanchez-Puerta M.V., Abbona C.C., Zhuo S., Tepe E.J., Bohs L., Olmstead R.G., Palmer J.D. (2011). Multiple recent horizontal transfers of the cox1 intron in Solanaceae and extended co-conversion of flanking exons. BMC Evol. Biol..

[69] Ishikawa N., Yokoyama J., Ikeda H., Takabe E., Tsukaya H. (2006). Evaluation of morphological and molecular variation in *Plantago asiatica* var. *densiuscula*, with special reference to the systematic treatment of *Plantago asiatica* var. *yakusimensis*. J. Plant Res..

[70] Ishikawa N., Yokoyama J., Tsukaya H. (2009). Molecular evidence of reticulate evolution in the subgenus *Plantago* (Plantaginaceae). Am. J. Bot..

[71] Stuessy T.F., Crawford D.J., López-Sepúlveda P. (2017). Plants of Oceanic Islands: Evolution, Biogeography, and Conservation of the Flora of the Juan Fernández (Robinson Crusoe) Archipelago.

[72] Matsuo K. (1989). Biosystematic studies on the genus *Plantago*. 1. Variations in *Plantago japonica* and its related species with special reference to its identity. Acta Phytotaxon. Geobot..

[73] Shipunov A. (2000). The genera *Plantago* L. and *Psyllium* Mill. (Plantaginaceae Juss.) in the flora of East Europe. Novit. Syst. Plant. Vasc..

[74] Palermo A.M., De Vita A., Peruzzi L., Gargano D., Bernardo L., Musacchio A. (2010). Does *Plantago brutia* Ten. (Plantaginaceae) merit specific rank? Insights from nrDNA and cpDNA data. Plant Biosyst..

[75] Tsinger N. (1905). *Plantago tenuiflora* W.K. and *Plantago minor* Fries. (to the question of the climate influence of the plant life and form). Mem. Soc. Nat. Kiew.

[76] Shipunov A. (2015). *Plantago schrenkii* is *P. maritima*: Morphological and molecular evidence. Ann. Bot. Fenn..

[77] Hassemer G. (2019). Mediterranean mysteries: Notes on *Plantago* sect. Lancifolia (Plantaginaceae). Phytotaxa.

[78] Meyen S.V. (1987). Fundamentals of Palaeobotany.

[79] Thiers B. Index Herbariorum: A Global Directory of Public Herbaria and Associated Staff. New York Botanical Garden’s Virtual Herbarium. http://sweetgum.nybg.org/science/ih.

