# Supplementary figures and images for: Molecular and Morphological Data Improve the Classification of Plantagineae (Lamiales)
Source: Plants (Basel). 2021 Oct 26;10(11):2299. doi: 10.3390/plants10112299 (PMC8625185; doi:10.3390/plants10112299)

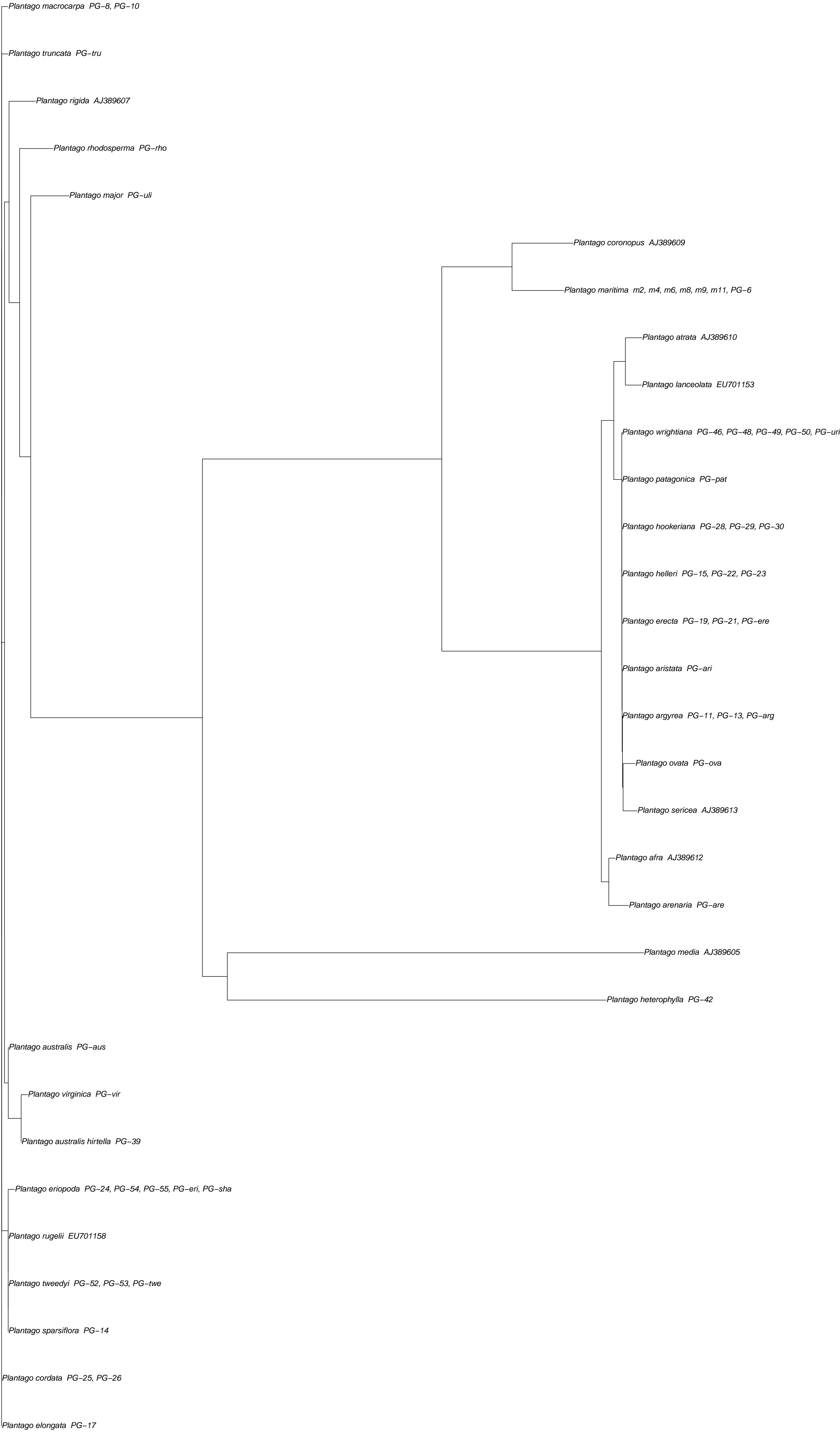

Supplement: Supplementary file 1 [file plants-10-02299-s001.zip › plants-1354731-supplementary/Figure S1.pdf]
